# The Toll pathway underlies host sexual dimorphism in resistance to both Gram-negative and Gram-positive bacteria in mated *Drosophila*

**DOI:** 10.1186/s12915-017-0466-3

**Published:** 2017-12-21

**Authors:** David F. Duneau, Hannah C. Kondolf, Joo Hyun Im, Gerardo A. Ortiz, Christopher Chow, Michael A. Fox, Ana T. Eugénio, J. Revah, Nicolas Buchon, Brian P. Lazzaro

**Affiliations:** 1 Université Toulouse 3 Paul Sabatier, CNRS, ENFA, UMR5174 EDB (Laboratoire Évolution & Diversité Biologique), 118 route de Narbonne, F-31062 Toulouse, France; 2 CNRS, Université Paul Sabatier, UMR5174 EDB, F-31062 Toulouse, France; 3 Present Address: Case Western Reserve University School of Medicine, Cleveland, Ohio USA; 4 Cornell Institute of Host Microbe Interactions and Disease, Cornell University, Ithaca, NY USA; 5 Instituto Gulbenkian de Ciência, Rua da Quinta Grande 6, P-2780 Oeiras, Portugal

**Keywords:** Sexual dimorphism, Innate immunity, Antimicrobial peptides, *Drosophila melanogaster*, Toll pathway

## Abstract

**Background:**

Host sexual dimorphism is being increasingly recognized to generate strong differences in the outcome of infectious disease, but the mechanisms underlying immunological differences between males and females remain poorly characterized. Here, we used *Drosophila melanogaster* to assess and dissect sexual dimorphism in the innate response to systemic bacterial infection.

**Results:**

We demonstrated sexual dimorphism in susceptibility to infection by a broad spectrum of Gram-positive and Gram-negative bacteria. We found that both virgin and mated females are more susceptible than mated males to most, but not all, infections. We investigated in more detail the lower resistance of females to infection with *Providencia rettgeri*, a Gram-negative bacterium that naturally infects *D. melanogaster*. We found that females have a higher number of phagocytes than males and that ablation of hemocytes does not eliminate the dimorphism in resistance to *P. rettgeri*, so the observed dimorphism does not stem from differences in the cellular response. The Imd pathway is critical for the production of antimicrobial peptides in response to Gram-negative bacteria, but mutants for Imd signaling continued to exhibit dimorphism even though both sexes showed strongly reduced resistance. Instead, we found that the Toll pathway is responsible for the dimorphism in resistance. The Toll pathway is dimorphic in genome-wide constitutive gene expression and in induced response to infection. Toll signaling is dimorphic in both constitutive signaling and in induced activation in response to *P. rettgeri* infection. The dimorphism in pathway activation can be specifically attributed to Persephone-mediated immune stimulation, by which the Toll pathway is triggered in response to pathogen-derived virulence factors. We additionally found that, in absence of Toll signaling, males become more susceptible than females to the Gram-positive *Enterococcus faecalis*. This reversal in susceptibility between male and female Toll pathway mutants compared to wildtype hosts highlights the key role of the Toll pathway in *D. melanogaster* sexual dimorphism in resistance to infection.

**Conclusion:**

Altogether, our data demonstrate that Toll pathway activity differs between male and female *D. melanogaster* in response to bacterial infection, thus identifying innate immune signaling as a determinant of sexual immune dimorphism.

**Electronic supplementary material:**

The online version of this article (doi:10.1186/s12915-017-0466-3) contains supplementary material, which is available to authorized users.

## Background

The most striking differences among individuals in a population are often those between the sexes. Such dimorphism is often characterized by obvious differences in morphology and behavior, as well as in a number of differences in physiological functions, including immunity, metabolism, and disease outcome [1]. The sex of the host influences both the dynamics of infections [2] and host symptoms [3], with a possible impact on parasite adaptation to the host when transmission is sex biased [4]. Despite the plethora of examples of sexual dimorphism in disease outcomes [3, 5, 6], the characterization of these differences have been largely overlooked in medical studies and in studies of natural systems [7–11]. Herein, we used *Drosophila melanogaster* to understand the basis of sexual dimorphism of an innate immune system.

*D. melanogaster* is a powerful model system with a well-defined innate immune response [12]. The cellular response to bacteria consists of defensive phagocytosis by specialized cells called plasmatocytes (functional equivalent of mammalian macrophages). The humoral response in adults is characterized by oxidative melanization and production of antimicrobial peptides (AMPs). The production of AMPs is regulated by two pathways, the Imd and the Toll pathways, which are homologous to the vertebrate TNF and Toll-like pathways, respectively [12]. The Imd pathway is activated upon the detection of peptidoglycan produced by Gram-negative bacteria, whereas the Toll pathway responds to the peptidoglycan of most Gram-positive bacteria and to proteases secreted during pathogenic infections [13]. Activation of each of these pathways leads to the nuclear translocation of transcription factors in the NF-κB family, driving a robust transcriptional response to infection [14, 15]. *D. melanogaster* exhibit sexual dimorphism in disease outcome. Recent studies have reported that males and females differ in response to gut [16] and systemic bacterial infection [17, 18], as well as to infection by pathogenic fungi [19, 20] and viruses [21]. However, the molecular basis behind this dimorphism remains unknown.

Here, we establish that infection with a broad spectrum of Gram-positive and Gram-negative bacteria results in a sexually dimorphic outcome in both outbred and inbred populations of mated *D. melanogaster*. This is not due only to the previously described [22–24] increased susceptibility of mated females. We dissect in detail the genetic and mechanistic basis for the dimorphic response to infection with *Providencia rettgeri*, a Gram-negative bacterial pathogen that naturally infects wild *D. melanogaster*. We find that the Toll signaling pathway is sexually dimorphic in genome-wide constitutive gene expression and in induced response to infection. Higher expression of Toll signaling genes and higher activity of Persephone-mediated Toll signaling explains the higher resistance of males relative to females.

## Methods

### Host husbandry and genotypes

*D. melanogaster* were reared until adulthood on glucose-yeast medium (100 g/L yeast, 100 g/L glucose, 1% agar). At day 2 after eclosion, adults were isolated in groups of five males and five females per vial. All experiments were conducted with mated individuals 5 to 8 days post-eclosion unless stated otherwise. Husbandry and experiments were conducted at 24 °C (±1 °C) with a 12 h:12 h light:dark cycle. We used different *D. melanogaster* genotypes – Canton-S (Bloomington stock # 1), Oregon R (Bloomington stock # 5), and w^1118^ (Bloomington stock #6326) as ‘wildtype’ laboratory strains and an outbred population that was derived from the Global Diversity Lines [25, 26] and kindly provided by J. Ayroles and A. Clark. To label phagocytes, we used the reporter lines *Hemolectin-Gal4 > UAS-GFP*, *eater-nls::GFP*, *Peroxidasin-Gal4 > UAS-GFP*, and *eater-Gal4 > UAS-dsred* [27–29]*.* To test the contribution of different components of innate immunity to sexual dimorphism in survival to infection, we used a series of null mutants, namely (1) *PPO1* ^*Bc*^ *fj* ^*1*^ *wt* ^*1*^ [30] and triple mutant PPO1^Δ^,2^Δ^,3^1^ [31, 32], which have deficient melanization, (2) *PGRP- LE* ^*112*^ *;+;PGRP-LC* ^*ΔE*^ [33], *imd* ^10191^ [34], *dTAK1* ^D10^ [35], and *Relish E20* [36], which have deficient Imd pathway function, and (3) *spz* ^rm7^/TM6C [37], *modSP* ^*α33*^ [38], and *psh* ^*1*^ [39], which have deficient Toll pathway function. To test the contribution of cellular immunity, we used phagocyte-depleted flies generated by inducing the pro-apoptotic gene *Bax* in Hml-expressing hemocytes (*Hml-Gal4 > UAS-GFP*, *UAS-bax*, *Gal80ts*) [40, 41]. We avoided potential deleterious developmental effects of phagocyte depletion by rearing the flies at 18 °C through pupation, and then switching freshly eclosed adults to 29 °C for 3 days, driving expression of the bax toxin. Phagocyte-depleted flies were then returned to 25 °C for 2 days before injection. Since there is no hematopoietic organ in *D. melanogaster* adults, these flies remained without phagocytes even after return to 25 °C (Additional file 1: Figure S4A).

### Bacterial strains and infection

We used a variety of bacterial strains, including both Gram-negative and Gram-positive bacteria known to vary in virulence in *D. melanogaster*. For Gram-negative bacteria, we used *Pectinobacterium carotovora carotovora* 15 (formerly known as *Erwinia carotovora carotovora 15*), *Ecc15*-GFP [42], and *Escherichia coli* (pEGFP, Mach1-T1 strain) with low virulence (i.e., does not kill at low dose), as well as strains of two *Providencia* species isolated from wild-caught *D. melanogaster* [43, 44], namely *P. rettgeri* (strain *Dmel* and its GFP-transformed derivative used in visualization experiments), which is moderately virulent (i.e., kills 80–50% of the flies infected at low dose), and *P. alcalifaciens* (strain *Dmel*), which is highly pathogenic (i.e., all flies infected at low dose die within a day). For Gram-positive bacteria, we used *Enterococcus faecalis*, a moderately virulent bacterium isolated from wild-caught *D. melanogaster* [45] and *Staphylococcus aureus* (PIG1), a highly virulent bacterium. Liquid cultures of each bacterium were grown to saturation overnight at 37 °C, except *Ecc15*, which was grown at 29 °C. Saturation cultures were pelleted, then resuspended and diluted in phosphate buffered saline (PBS, pH 7.4) to an optical density (OD) of 0.1 (600 nm wavelength) unless stated otherwise. We injected 23 nL of bacterial suspension into each fly abdomen using a Nanoject II (Drummond), corresponding to a dose of approximately 3000 viable bacteria per fly (for OD = 0.1). Injection of the same volume of sterile PBS was used as a wounding treatment. Flies injected with PBS experienced minimal to no mortality during the experiments. Flies were anesthetized with CO_2_ for less than 5 min during the injection procedure, and then were observed shortly after injection to confirm recovery from manipulations. The number of individuals and of experimental repetitions are indicated by ‘df +1’ for the factor ‘Experiment’ in each analysis reported in the figure legends.

### Host survival

We monitored host survival after injection in groups of 50 males and 50 females kept together in 900-mL plastic boxes with ad libitum access to food, monitoring survival daily for 10 days unless otherwise specified. We focused our study on the first 10 days of the infection to avoid an interaction between the effect of ‘sex’ and ‘age’ in response to infection. Individuals alive after 10 days were recorded as censored observations. In experiments where sexual dimorphism was compared between genetic lines, injected individuals were kept solitary in vials to avoid potential confounding effects of density due to differential rates of mortality among the groups. Host survival differences were analyzed using a Cox’s proportional hazards mixed model, with ‘Sex’ and ‘Dose’ as main effects and ‘Replicate’ as random effect using the R package ‘Survival’ [46]. The cox.zph function from the same package was used to ensure that the data fit the assumptions of the proportional hazards approach.

### Bacterial load

To monitor bacterial loads, flies were individually homogenized in 500 μL of sterile PBS with a HT homogenizer (OPS Diagnostics). The homogenate was then diluted 1:10 in PBS and 50 μL of this suspension was plated onto LB agar using a WASP II autoplate spiral plater (Microbiology International). Plates were incubated overnight at 37 °C (endogenous microbiota do not appear on LB plates during this time) and bacterial colonies were counted using a EZ-Count Automated Colony Counter (Microbiology International) to estimate the number of viable bacteria per fly. Bacterial load differences were compared with a Kruskal–Wallis or a Wilcoxon test when possible. Because non-parametric tests can be underpowered, we additionally performed a parametric Welsh *t* test. When ‘Time post-injection’ and ‘Line’ were included in the analysis, we used a linear model assuming a Gaussian distribution of the error. The residual error was checked for normality and homoscedasticity, and when necessary the raw data were subjected to Box–Cox transformation. To include ‘Replicate’ as a random effect, we used a generalized linear mixed model with the function HLfit from the R package ‘spaMM’ [47]. In that case, *P* values were obtained from model selections and likelihood ratios (LR2) were given.

### Hemolymph quantification

To estimate the quantity of hemolymph in male and female hosts, we weighed 3- to 5-day-old flies individually on a Mettler Toledo (MX5) microbalance to the nearest microgram, before and after removing the hemolymph. We removed the hemolymph by separating the thorax and abdomen of individual flies and absorbing the hemolymph by contact with a Kimwipe (Kimberly Clark) as previously described [48].

### Phagocyte counts and phagocytosis

In order to quantify hemocytes, *Hml-Gal4 > UAS-GFP*, *eater-nlsGFP*, and *Pxn-Gal4 > UAS-GFP* flies were decapitated and injected with sterile PBS (Nanoject II; Drummond) to detach sessile phagocytes from the tissues. Groups of 5 or 15 same-sex flies were then bled onto a glass slide, which was imaged on a Zeiss microscope (axioplot imager Zeiss) [49]. For each slide, the number of phagocytes per 25 mm^2^ was counted using Zeiss’ Zen software. We analyzed the difference in phagocyte numbers between males and females with paired Wilcoxon tests, pairing males and females of the same genotype and manipulating them in parallel. To estimate the proportion of phagocytes actively engaged in phagocytosis, we injected flies with 23 nL of 1 mg/mL pHrodo-labeled dead *E. coli* (Molecular Probes, cat# P35361), which becomes fluorescent upon phagosome maturation, and counted the number of phagocytes containing labeled bacteria using the same procedure. To confirm that *P. rettgeri* was phagocytized by phagocytes, we injected bacteria carrying a plasmid expressing GFP into host flies expressing dsRed specifically in phagocytes (*eater-Gal4 > UAS-dsred*). We recorded the phagocytosis events with confocal microscopy (axioplot imager Zeiss).

### Estimation of the time before control of bacterial proliferation (${\overline{t}}^c$)

We estimated the averaged time required for the host to control bacterial proliferation (${\overline{t}}^c$) as performed previously [50]. In short, we built a model that consists of a mixture of three models, one describing within-host bacterial growth, one modeling bacterial reduction if the host controls the infection, and one modeling the likelihood that a given fly controls or fails to control the infection. These models were then fit to empirical data of bacterial growth trajectories in males and females, using eight individually plated flies per hour post-injection to estimate *t* ^*c*^ for each sex. Confidence intervals for parameter estimates were obtained by bootstrapping the empirical data for each time point and re-estimating model parameters.

### RNA isolation and library construction

We measured the whole transcriptional response in male and female Canton S 8 h after the infection started. Flies were injected with approximately 3000 bacteria in suspension in 23 nL of PBS as described above. A subset of the flies was dissected to remove their reproductive tract prior to RNA isolation. Each sample was a same-sex pool of 25 flies homogenized in 1 mL of TRIzol (Life Technologies). Unchallenged flies were simultaneously treated the same way as injected flies, except that they were only exposed to CO_2_ and did not receive an injection. We quantified expression in biological triplicates of unchallenged and infected pools. Six additional pools of 25 unchallenged flies for each sex were added to estimate the constitutive expression of the Toll pathway. Those additional pools were exposed to CO_2_ at the same time as the initial pools but were homogenized 24 and 72 h after being anesthetized. RNA and library quality was determined by AATI Fragment Analyzer. Libraries were prepared using the Lexogen Quantseq 3’ mRNA-Seq Library Prep kit, following manufacturer’s instructions. Sequencing was performed on the Illumina Hiseq 2500 Rapid Run Mode platform at the Cornell Life Sciences Sequencing Core, with 50 bp single-end reads.

### Read mapping, normalization and quantification of expression differences

For each sample, at least 5 million sequence reads were generated. Read quality was estimated by fastqc for quality control and reads were trimmed using Trimmomatic ([51], version 0.32). Trimmed reads were then mapped to the *D. melanogaster* reference genome (version 6.80), using STAR RNA-seq aligner ([52], version 2.4.1a). Counts were calculated using ‘htseq’ ([53], version 0.6.1). We characterized the difference between males and females in transcriptional response to infection by the interaction between the treatment (unchallenged vs. infected) and the sex (male vs. female). The model was then Number of reads = Treatment + Sex + Treatment*Sex. We performed the analysis with the R package ‘DEseq2’ [54] and visualized the significant candidates (i.e., *P* value lower than 0.05 after an FDR correction) in volcano plots using the R package ggplot2 [55]. Heatmap figures were generated with the package ‘Heatmap3’ with default settings to calculate the clustering [56]. Gene ontology (GO) enrichment analysis was performed with the software Gorilla (available at http://cbl-gorilla.cs.technion.ac.il/) to search for GO categories that are enriched in the ‘target’ set (herein, the list of female genes differently expressed in infected host compared to non-infected) compared to the ‘background’ set (herein, the list of male genes differently expressed in infected host compared to non-infected) using the standard Hyper Geometric statistics [57].

### Quantification of host gene expression by RT-qPCR

To evaluate Toll pathway activity, we used qPCR to measure the expression of *Drosomycin*, an antimicrobial peptide gene strictly regulated by the Toll pathway [13], using primers CGTGAGAACCTTTTCCAATATGATG (forward) and TCCCAGGACCACCAGCAT (reverse). To evaluate Imd pathway activity, we measured the expression of *Diptericin*, an antimicrobial peptide gene strictly regulated by the Imd pathway [13] using primers GCGGCGATGGTTTTGG (forward) and CGCTGGTCCACACCTTCTG (reverse). Inter-sample variation in cDNA content was controlled by analysis of reference gene *RpL32* (Forward primer: GACGCTTCAAGGGACAGTATCTG, Reverse primer: AAACGCGGTTCTGCATGAG), which has been shown to not respond to infection [58]. We obtained total RNA from groups of eight to ten flies at 24 and 72 h after injection of *P. rettgeri* or PBS. In a second experiment, we obtained the total RNA from groups of four flies at 8 h after injection of *P. rettgeri* (before any death happened) or unchallenged control individuals. Flies were homogenized in 1 mL of TRIzol (Life Technologies), immediately stored at –80 °C, and RNA was isolated using a standard protocol. One microgram of DNase-treated (Promega) RNA was reverse-transcribed using MLV-RT (Promega). The cDNA thus produced served as template for quantitative PCR using the SSO Advanced SYBR Green Kit (Bio-Rad) under minor modification of the manufacturer’s instructions.

## Results

### Sexual dimorphism in host susceptibility to infection is pervasive

**Fig. 1:**
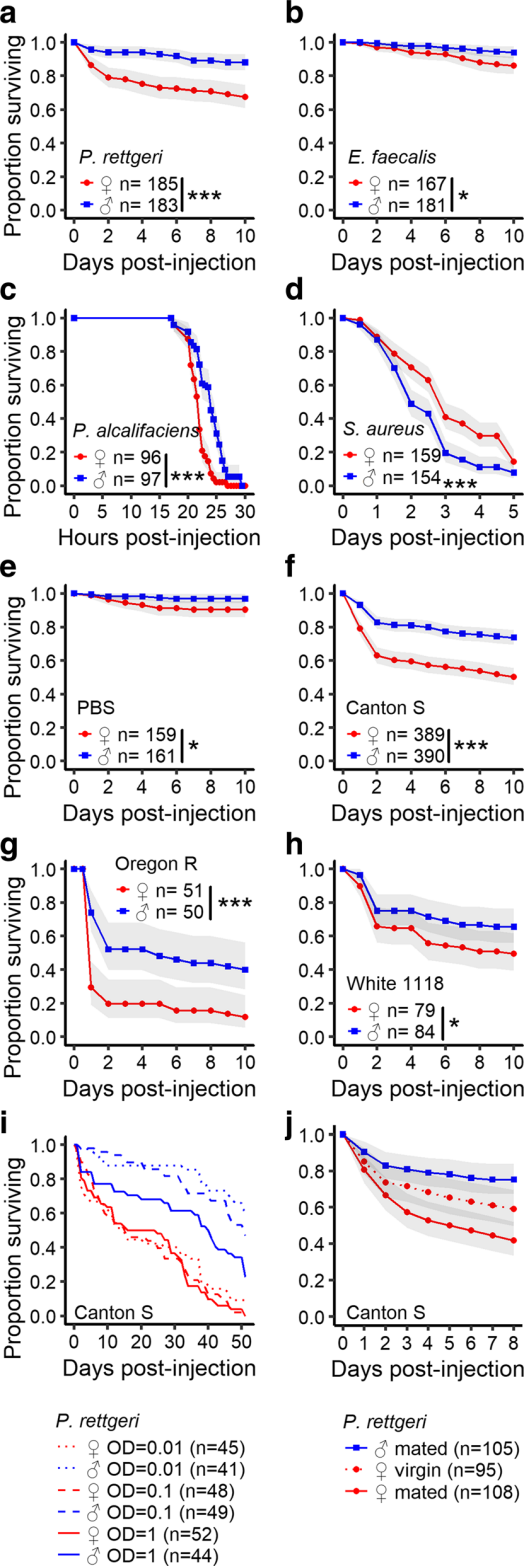
Sexual dimorphism in survival to infection of an outbred population. **a** Survival upon infection with the Gram-negative bacteria *P. rettgeri*. Females were more susceptible than males (Cox-ph: df = 1, χ^2^ = 23.26, *P* < 0.0001). **b** Survival upon infection with the Gram-positive bacteria *Enterococcus faecalis*. Females were more susceptible than males (Cox-ph: df = 1, χ^2^ = 5.96, *P* = 0.015). **c** Survival upon infection with the Gram-negative bacteria *P. alcalifaciens*. Females were more susceptible than males (Cox-ph: df = 1, χ^2^ = 42.49, *P* < 0.0001). **d** Survival upon infection with the Gram-positive bacteria *Staphylococcus aureus*. Males were more susceptible than females (Cox-ph: df = 1, χ^2^ = 7.37, *P* = 0.006). **e** Survival upon injection of *PBS* (sterile solution used for bacterial suspension). Females were slightly more susceptible than males (Cox-ph: df = 1, χ^2^ = 5.66, *P* = 0.02). **f** Survival of the genotype Canton S upon infection by the Gram-negative bacteria *P. rettgeri*. Females were more susceptible than males (Cox-ph: *Sex*: df = 1, χ^2^ = 49.42, *P* < 0.0001). **g** Survival of the genotype Oregon R upon infection by the Gram-negative bacteria *P. rettgeri*. Females were more susceptible than males (Cox-ph: *Sex*: df = 1, χ^2^ = 16.09, *P* < 0.0001). **h** Survival of the genotype *w* ^1118^ upon infection by the Gram-negative bacteria *P. rettgeri*. Females were more susceptible than males (Cox-ph: *Sex*: df = 1, χ^2^ = 4.36, *P* = 0.036). **i** Dose response in survival of Canton S to *P. rettgeri* infection. Females were more susceptible than males (Cox-ph: *Sex*: df = 1, χ^2^ = 96.97, *P* < 0.0001) and the dose had an effect on survival (*Dose*: df = 1, χ^2^ = 8.09, *P* = 0.004). This effect was dependent on the host sex as a 100-fold difference in starting dose was not enough to remove the dimorphism in survival (Dose*Sex: df = 1, χ^2^ = 5.64, *P* = 0.017). **j** Survival of mated males and females and virgin females upon infection with the Gram-negative bacteria *P. rettgeri*. Mated males are more resistant than mated females (Cox-ph adjusted for multiple tests: *Sex*: df = 1, z = 4.6, *P* < 0.001) and virgin females (Cox-ph adjusted for multiple tests: *Sex*: df = 1, z = 2.34, *P* = 0.045). Mated and virgin females were marginally non-significant in difference in survival (Cox-ph adjusted for multiple tests: *Sex*: df = 1, z = 2.31, *P* = 0.054)

In order to test whether *D. melanogaster* are sexually dimorphic in survivorship in response to infection, we first injected a genetically diverse population as well as several inbred populations of *D. melanogaster* with both Gram-negative (*P. rettgeri* and *P. alcalifaciens*) and Gram-positive (*E. faecalis* and *S. aureus*) bacteria that vary in virulence. In all cases, we found significant differences in survival between males and females from this outbred population (Fig. 1a–d). Females were generally more likely than males to die from infection and from an injection wound (Fig. 1e), irrespective of wildtype genotype (Fig. 1f–h and Additional file 2: Figure S1A) and initial bacterial dose (Fig. 1i and Additional file 2: Figure S1B, C). Interestingly, however, the sex dimorphism in host susceptibility was reversed after infection with *S. aureus* (Fig. 1d). This indicates that females are not globally weaker but rather that they are specifically more susceptible to most bacterial pathogens. We considered that the females in our study might be immunocompromised, as mating and reproduction have previously been shown to suppress immunity in *D. melanogaster* females [22–24]. To test this hypothesis, we compared the survival of mated and virgin females to mated males after infection with *P. rettgeri*. The mortality of virgin females was intermediate between those of mated males and of mated females (Fig. 1j). This result suggests that female mating status contributes to the sexual dimorphism in survival but is not sufficient to completely account for the effect. To further characterize host sexual dimorphism in susceptibility, we focused on a natural pathogen of *Drosophila* that shows moderate levels of virulence, the Gram-negative bacterium *P. rettgeri*.

### Females are less efficient at controlling pathogen growth

**Fig. 2:**
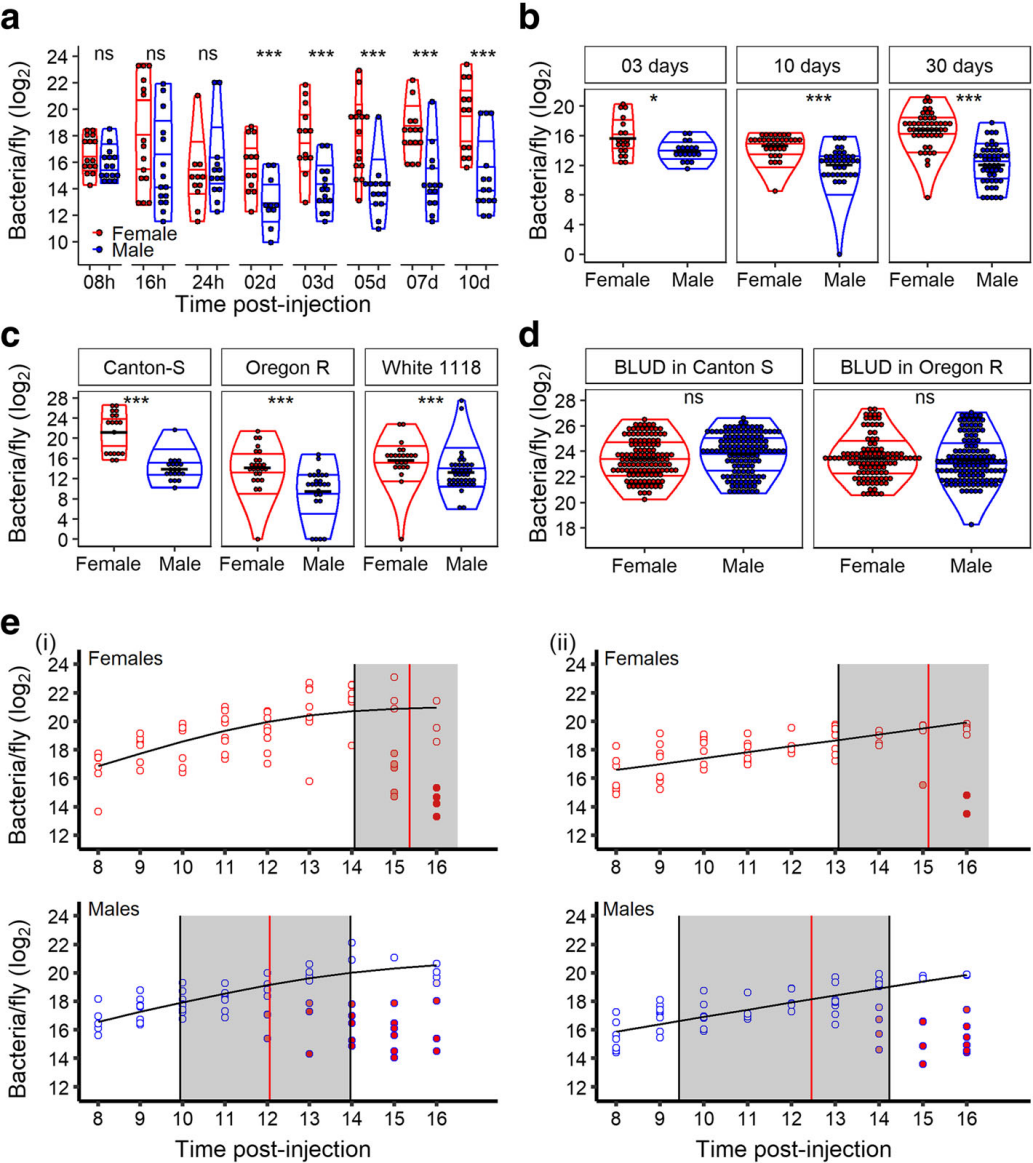
Sexual dimorphism in resistance to *P. rettgeri*. **a** *P. rettgeri* load in Canton S (CS) flies over 10 days of infection. Overall, females carried more bacteria than males. We did not detect differences in bacterial load in early time points (Wilcoxon test: 8 h: W = 151.5, *P* = 0.1102; 16 h: W = 151, *P* = 0.1148; 24 h: W = 60, *P* = 0.52). However, after 48 h post-injection, when the mortality plateaued and the infection can be considered chronically persistent, females carried a higher burden than males (Wilcoxon test: 2 days: W = 99.5, *P* = 0.01; 3 days: W = 171.5, *P* = 0.0007; 5 days: W = 202.5, *P* = 0.0002; 7 days: W = 188, *P* = 0.0003; 10 days: W = 152, *P* = 0.0005). **b** *P. rettgeri* load upon injection in flies from an outbred population. Females carried more bacteria than males during the chronic phase of infection of *P. rettgeri* in the three independent experiments looking at three different time points of the infection (Wilcoxon test: 3 days: W = 262.5, *P* = 0.01; 10 days: W = 1162, *P* < 0.0001; 30 days: W = 2148, *P* < 0.0001). **c** *P. rettgeri* load 10 days post-injection in flies from wildtype genotypes. For the three genotypes, females carried more bacteria than males during the chronic phase of infection of *P. rettgeri* (Wilcoxon test: CS: W = 350, *P* < 0.0001; w^1118^: W = 692.5, *P* = 0.0001; Oregon R (OrR): W = 513, *P* = 0.0001). **d** Bacterial load upon death (BLUD; +30 min maximum) in both sexes of CS and OrR flies. We did not detect a significant sexual dimorphism in *P. rettgeri* load at death (Welsh *t* test: CS: df = 255.28, *t* = –1.77, *P* = 0.08; OrR: df = 235.58, *t* = 0.6, *P* = 0.54). Both sexes succumbed at the same bacterial load. Black lines in b, c, and d represent the means. The violin plots represent the distribution of the data, the quartiles and the median. Wilcoxon tests comparing medians: *P < 0.05, ****P < 0.0001, and ns: *P* > 0.05. **e** Within-host dynamic of *P. rettgeri* in males and female CS flies between 8 and 16 h post-injection. In both experiment replicates (*i* and *ii*), males have controlled the bacterial proliferation before females (estimated *t* ^*c*^, or time of control, is represented by the vertical red lines and the confidence interval around the estimate is shaded in gray). Each dot represents the bacterial load in a single fly, the solid line represents the standard Baranyi bacterial population growth fitted to the white dots (see Methods and [50]). The intensity of red in the dots represents the inferred probability that the host controlled the infection (see Methods and [50])

To elucidate whether males and females differ in their ability to control pathogen proliferation, we infected males and females with *P. rettgeri* and quantified the bacterial load in each individual (Fig. 2). We first monitored the sexual dimorphism in bacterial burden in a wildtype (Canton S) host at different time points of the infection (Fig. 2a) and determined that the burden seems to stabilize between 2 and 3 days post-injection, at the same time when host mortality plateaued (Fig. 1f). Surviving flies sustain persistent infection and do not eliminate the bacteria. While we did not detect a sexual dimorphism in resistance before the chronic persistence phase (8, 16, and 24 h), females carried more than 10 times the bacterial load during the chronic phase of infection (3, 5, 7, and 10 days; Fig. 2a). This difference in bacterial load during the chronic phase of the infection was also observed in our outbred population at 3, 10, and 30 days post-injection (Fig. 2b) and in other inbred wildtype genotypes at 10 days post-injection (Fig. 2c). In order to make sure that the difference in bacterial burden was not due to the sexual dimorphism in body size, we weighed the total mass and the hemolymph of males and females from three wildtype genotypes (Additional file 3: Figure S2). Females were only approximately 20% larger than males and had about twice the quantity of hemolymph as males, whereas the difference in bacterial load was 10-fold. Thus, bacterial load does not scale as a simple function of fly size. We have recently shown that flies die at a fixed bacterial burden, called the Bacterial Load Upon Death (BLUD) [50], which is the maximum number of bacteria a fly can sustain before dying. We quantified the BLUD for males and females by checking infected hosts every 30 min and determining the bacterial load in newly dead flies at each observation point. We did not detect sexual dimorphism in BLUD of either Canton S or Oregon R genotypes (Fig. 2d), suggesting that males and females die at a similar bacterial burden.

We then focused on the initial phase of infection to better understand the correlation between bacterial proliferation within the host and sexual dimorphism in survival. We previously showed that the survival outcome of infection with *P. rettgeri* for each individual depends of the time at which the immune response becomes effective and can control bacterial proliferation (time to control) [50]. Using our previously published mixture model and a bootstrap approach on hourly bacterial load data from the 8th to the 16th h after injection (see Methods), we estimated the average time to control ${\overline{t}}^c$ in males and females (Fig. 2e). Individual male flies were more likely to control the infection (illustrated as red dots in Fig. 2e) as a population, and males started to control infection earlier than females did (Fig. 2e). We found that the ${\overline{t}}^c$ differed by 2–3 h between males and females, with little or no overlap between the confidence intervals on ${\overline{t}}^c$ estimation, corresponding to a difference of roughly 5–8 bacterial doublings [43] prior to control.

Our data suggested that the sexual dimorphism in susceptibility to infection was due to sexual dimorphism in within-host bacterial growth. We considered that differences in bacterial growth might be due to a difference in resources available to bacteria infecting males and females. To investigate this, we injected *P. rettgeri* into living hosts or into hosts that were killed by decapitation either at the moment of the injection or 1 h before the injection. Since dead hosts are unable to mount an inducible immune response, we reasoned that sexual dimorphism in bacterial growth would be maintained in dead hosts if it was due to differences in resources available to the pathogen within male and female hosts, but lost if the dimorphic response was due to inducible responses. We did not see any difference in bacterial growth within dead male and female hosts, despite the sexual dimorphism in living hosts (Additional file 4: Figure S3). We therefore concluded that that the sexual dimorphism was due to a difference in active control of the proliferation, such as by the innate immune response.

### Cellular immunity and melanization are not responsible for sexual dimorphism in response to *P. rettgeri*

**Fig. 3:**
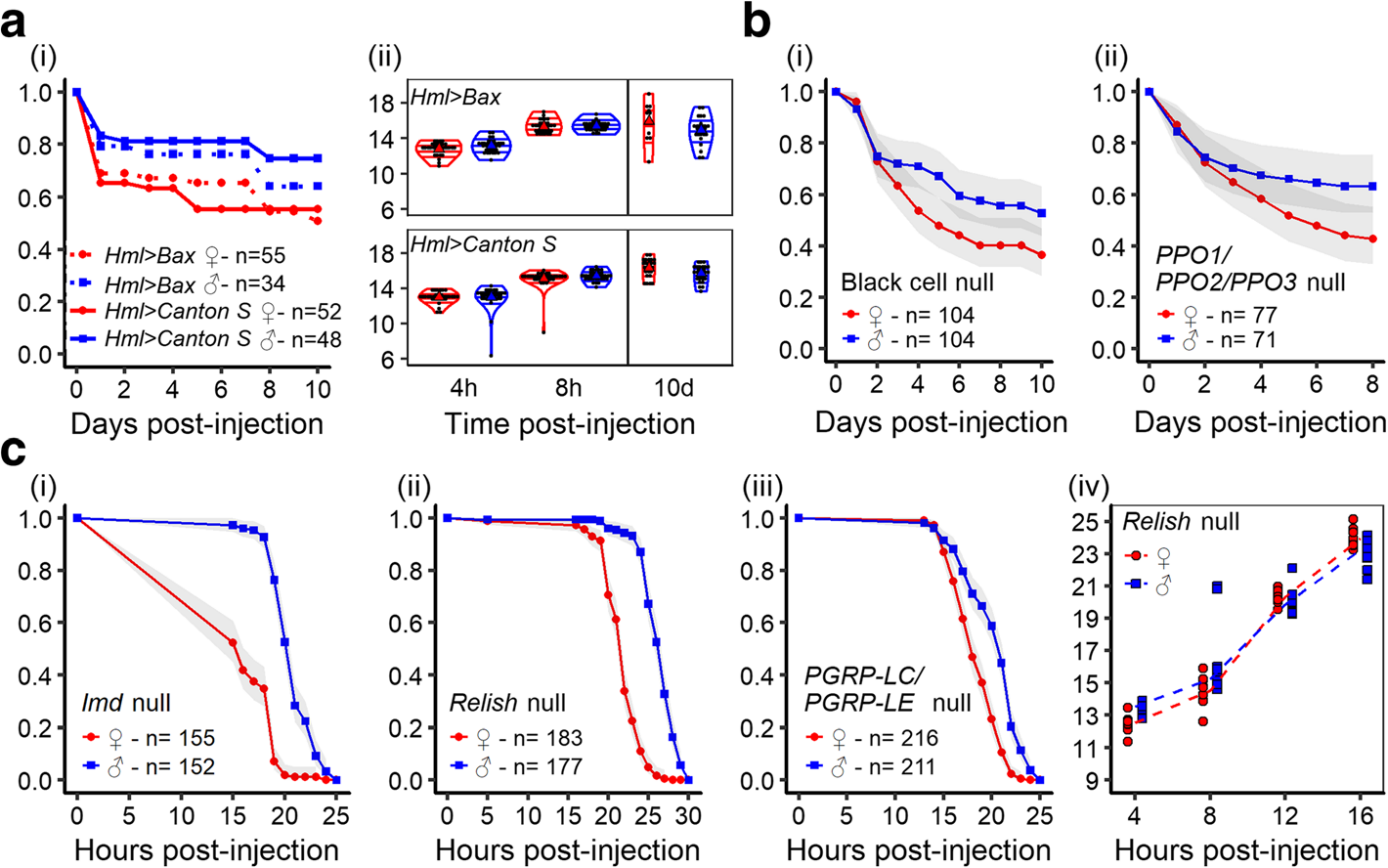
Phagocytosis, melanization, and the Imd pathway do not explain the sexual dimorphism. **a** The sexual dimorphism in survival to *P. rettgeri* is still present when hosts are not able to phagocytose bacteria. i Survival of flies lacking phagocytes compared to their genetic control after infection by *P. rettgeri* (OD = 0.1). Females of both lines were more susceptible than males and the lack of phagocytes did not affect the probability of surviving the infection (Cox-ph: Line: df = 1, χ^2^ = 1.01, *P* = 0.32; *Sex*: df = 1, χ^2^ = 18.41, *P* = 0.02; Line*Sex: df = 1, χ^2^ = 0.45, *P* = 0.50). ii *P. rettgeri* growth in flies lacking phagocytes and in their genetic control. Even if *P. rettgeri* was phagocytized, the bacterial growth was not significantly affected by this immune response neither in early time points (Linear regression: *Line*: df = 1, F = 0.28, *P* = 0.59; *Time*: df = 1, F = 577.82, *P* < 0.0001; *Sex*: df = 1, F = 3.63, *P* = 0.05; Time*Sex: df = 1, F = 0.0003, *P* = 0.99; Line*Sex: df = 1, F = 0.039, *P* = 0.84), nor at 10 days post-injection (Linear regression: *Line*: df = 1, F = 0.62, *P* = 0.43; *Sex*: df = 1, F = 20.36, *P* < 0.0001; Line*Sex: df = 1, F = 1.07, *P* = 0.3). **b** The sexual dimorphism in survival to *P. rettgeri* is still present when hosts are not able to melanize bacteria. i Black cell loss-of-function mutant (Cox-ph: *Sex*: df = 1, χ^2^ = 5.15, *P* = 0.02). ii Triple mutant PPO1^Δ^, 2^Δ^,3^1^ loss-of-function (Cox-ph: *Sex*: df = 1, χ^2^ = 4.46, *P* = 0.03). **c** The sexual dimorphism in survival to *P. rettgeri* is still present when hosts are not able to induce an Imd response against bacteria. Females were more susceptible to *P. rettgeri* than males for the four mutants tested. i *Imd* loss-of-function mutant (Cox-ph: *Sex*: df = 1, χ^2^ = 156.24, *P* < 0.0001). ii *Relish* loss-of-function mutant (Cox-ph: *Sex*: df = 1, χ^2^ = 230.99, *P* < 0.0001). iii Double loss-of-function mutant *PGRP-LC*, *PGRP-LE* (Cox-ph: *Sex*: df = 1, χ^2^ = 71.9, *P* < 0.0001). iv *P. rettgeri* growth in *Relish* loss-of-function mutant. The infection started with the same number of bacteria in both sexes (Linear regression: *Sex*: df = 1, F = 1.02, *P* = 0.32). The bacterial populations have grown over time (*Time*: df = 1, F = 766.96, *P* < 0.0001) but the growth was significantly different between sexes (Time*Sex: df = 1, F = 5.32, *P* = 0.02). Imd response does not explain the sexual dimorphism in susceptibility to *P. rettgeri*

Hemocytes perform defensive phagocytosis and promote resistance to pathogens [40, 59, 60]. We thus asked whether sexual differences in phagocyte counts and in phagocytosis efficiency could underlie the sexually dimorphic response to *P. rettgeri*. We first confirmed that *P. rettgeri* can be phagocytized by injecting GFP-labeled *P. rettgeri* into flies expressing dsRed in their phagocytes (eater-dsRed) and observing co-localization of green and red fluorescence under confocal microscopy (Additional file 1: Figure S4B). We observed that females of three different *D. melanogaster* genotypes that express GFP specifically in their phagocytes had more phagocytes than males (Additional file 1: Figure S4C, left panel), which is the opposite of what would be expected if differential phagocytosis was responsible for the dimorphism. We then tested whether males and females differed in phagocytosis efficiency, using dead pHrodo-labeled *E. coli* that fluoresce red only when in the low-pH phagolysosome compartment. We found no difference in the number of phagocytes with bacteria in their phagolysosome compartment (Additional file 1: Figure S4C, right panel), indicating no difference in the capability for phagocytosis. Finally, we directly tested the requirement for phagocytes in host survival of *P. rettgeri* infection by measuring survival (Fig. 3Ai) and bacterial load (Fig. 3Aii) of wildtype flies compared to flies whose hemocytes had been genetically ablated at the adult stage. We saw little effect of phagocyte ablation on survivorship of infection or on systemic pathogen load in either sex and the dimorphism persisted even in the absence of phagocytes (Fig. 3A). These collective results suggest that phagocytosis is not the key immune response that generates immune sexual dimorphism.

We next tested the role of immune-regulated melanization [31] as potentially contributing to dimorphism in susceptibility to *P. rettgeri* infection. We compared survival between males and females in the melanization-deficient mutant ‘Black cells’ and in a fly deficient for all three prophenoloxidases (key enzymes for melanization). In both cases, females deficient for melanization were more susceptible to infection than deficient males (Fig. 3B), suggesting that melanization does not play a role in the dimorphism. Altogether, our results show that the sexual dimorphism in resistance to *P. rettgeri* infection is not due to differences in cellular immunity or immune melanization between males and females.

### Imd pathway is central to controlling *P. rettgeri* growth but is not responsible for the dimorphism in susceptibility

Gram-negative bacteria induce the Imd pathway, which is critical for surviving these infections [13]. Furthermore, Imd pathway-regulated genes have been shown to have higher expression in *D. melanogaster* males than in females [61]. We therefore tested whether the Imd pathway is responsible for the observed sexual dimorphism in susceptibility to *P. rettgeri* infection by infecting several mutants that are deficient in Imd pathway activity with *P. rettgeri* and monitoring their survival. We used loss-of-function mutants of multiple genes involved in the Imd pathway, namely *Imd* (*Imd* ^*10191*^) [34], *Relish* (*Rel* ^*E20*^) [36], *dTAK1* (*dTAK1* ^*D10*^) [35], and a double mutant for both *PGRP-LC* and *PGRP-LE* (*PGRP- LE* ^*112*^;+;*PGRP-LC* ^*ΔE*^) [33]. Separately testing this complete set of mutants has the effect of querying the pathway at multiple points and of assessing the role of the Imd pathway in multiple genetic backgrounds. All of the mutant strains were much more susceptible to *P. rettgeri* infection than wildtype strains, with 100% of the mutant individuals dying in less than 48 h, thus confirming that the Imd pathway is absolutely required to fight *P. rettgeri* infection. Nevertheless, for all the Imd mutants tested, female flies still died significantly more quickly than males and the dimorphism was preserved (Fig. 3C and Additional file 5: Figure S5A). Interestingly, the exponential growth of *P. rettgeri* in Imd-deficient hosts over 16 h was also faster in females than in males (Fig. 3Civ) and sexual dimorphism was present in bacterial load at 8 and 12 h post-injection in *Relish* mutants (Additional file 5: Figure S5B). This suggests that, in absence of the Imd pathway, the hosts still retain some ability to control the infection and that ability is sexually dimorphic. Thus, we conclude that, while the Imd pathway is important for defense against *P. rettgeri* infection, it does not underlie the observed sexual dimorphism.

### Difference in Toll pathway induction causes sexual dimorphism in susceptibility

**Fig. 4:**
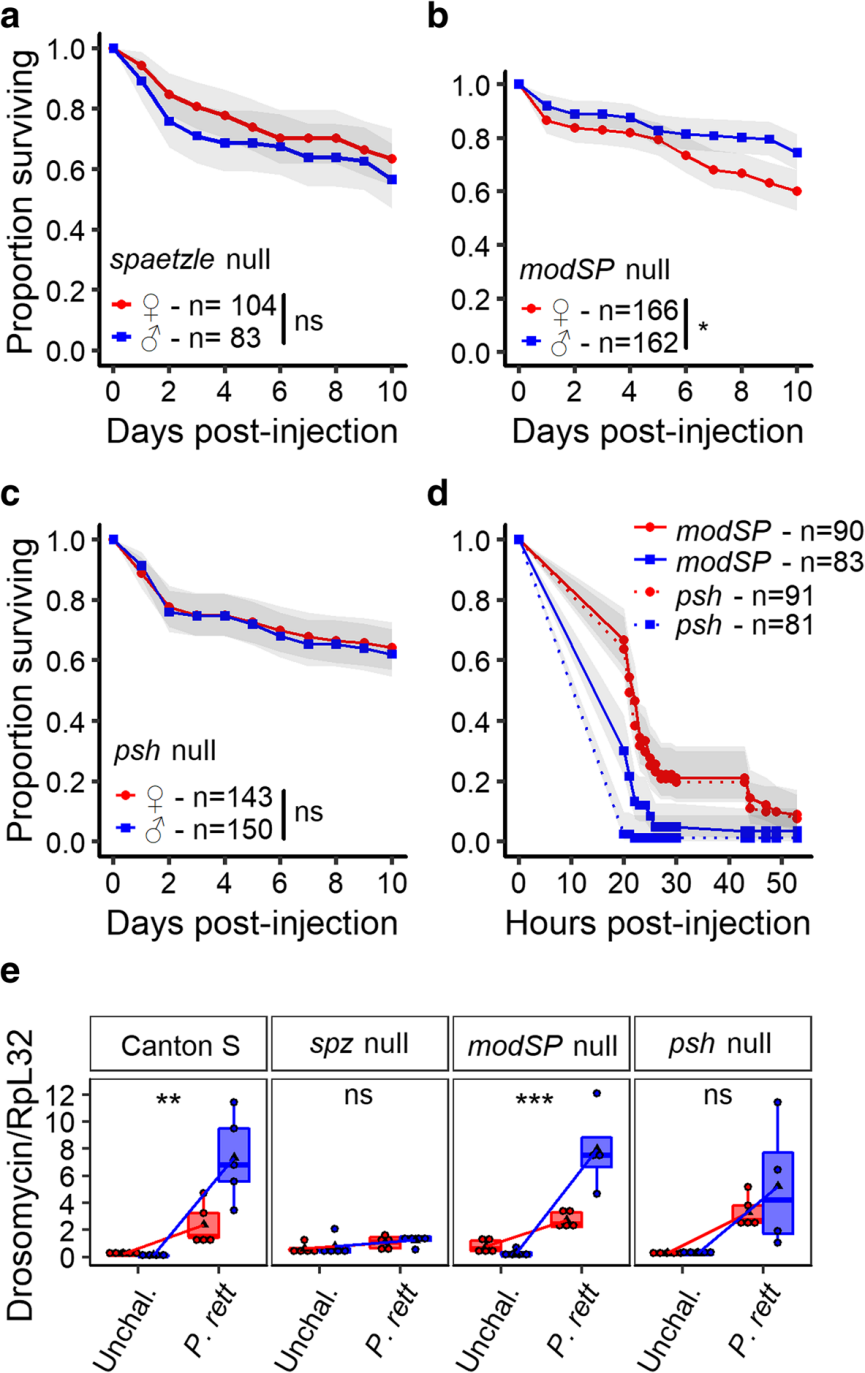
The Toll pathway is responsible for the sexual dimorphism in survival. **a** Sexual dimorphism in susceptibility in a loss-of-function mutant for the Toll pathway (*spaetzle* mutants). The probability of dying from *P. rettgeri* infection was the same for female and male hosts (Cox-ph: *Sex*: df = 1, χ^2^ = 1.12, *P* = 0.29). **b** Sexual dimorphism in susceptibility in loss-of-function mutant for the ModSP branch of the Toll pathway. Females were more susceptible than males (Cox-ph: *Sex*: df = 1, χ^2^ = 7.66, *P* = 0.005). Recognition of *P. rettgeri* by the ModSP component of the Toll pathway is not the reason for the sexual dimorphism in susceptibility. **c** Sexual dimorphism in susceptibility in loss-of-function mutant for the Persephone branch of the Toll pathway. The probability of dying from *P. rettgeri* infection was not different between female and male hosts (Cox-ph: *Sex*: df = 1, χ^2^ = 0.11, *P* = 0.74). The recognition of the virulence factor of the pathogenic Gram-negative bacteria *P. rettgeri* by the Toll pathway allows the sexual dimorphism. **d** Sexual dimorphism in susceptibility in loss-of-function mutant for the ModSP branch and for the Persephone branch of the Toll pathway. Females were less susceptible to *E. faecalis* infection than males for both mutant lines; males, but not the females, mutant for the Persephone branch where likely to die earlier than males mutant for the ModSP branch (Cox-ph: Line: df = 2, χ^2^ = 3.71, *P* = 0.05; *Sex*: df = 1, χ^2^ = 72.45, *P* < 0.0001; *Line/Sex*: df = 2, χ^2^ = 7.46, *P* = 0.006). Recognition of host damage or virulence factors is likely to be more important than the direct recognition of pathogenic bacteria. **e** Relative expression of *Drosomycin* to *RpL32* in both sexes in response to the infection by *P. rettgeri*. Toll response (i.e., difference in *Drosomycin* expression between unchallenged and infected) was stronger 8 h post-injection in male wildtypes and mutants for m*odSP* (Interaction *Sex* x *Treatment*: Canton S: df = 1, F = 13.68, *P* = 0.002; *modSP*: df = 1, F = 17.18, *P* = 0.0048) but this dimorphism was not present in mutants of *Persephone* and *spaetzle* (*psh*: df = 1, F = 0.35, *P* = 0.46; *spz*: df = 1, F = 0.03, *P* = 0.86)

The Toll pathway is canonically thought to underlie resistance to Gram-positive bacteria and fungi [12, 13], but can also be activated upon infection with pathogenic Gram-negative bacteria [62] and upon wounding [63–66]. To test whether the Toll pathway contributed to sexual dimorphism in immunity, we infected null mutants of *spaetzle* (*spz*) [37], which encodes the ligand for Toll in *Drosophila*, and monitored survival of both males and females. Null mutants of *spz* exhibited a mild susceptibility to *P. rettgeri* infection, but more importantly, they exhibited no sexual dimorphism in survivorship (Fig. 4a). This result indicates that a functional Toll pathway is required for the dimorphic susceptibility to *P. rettgeri* infection.

**Fig. 5:**
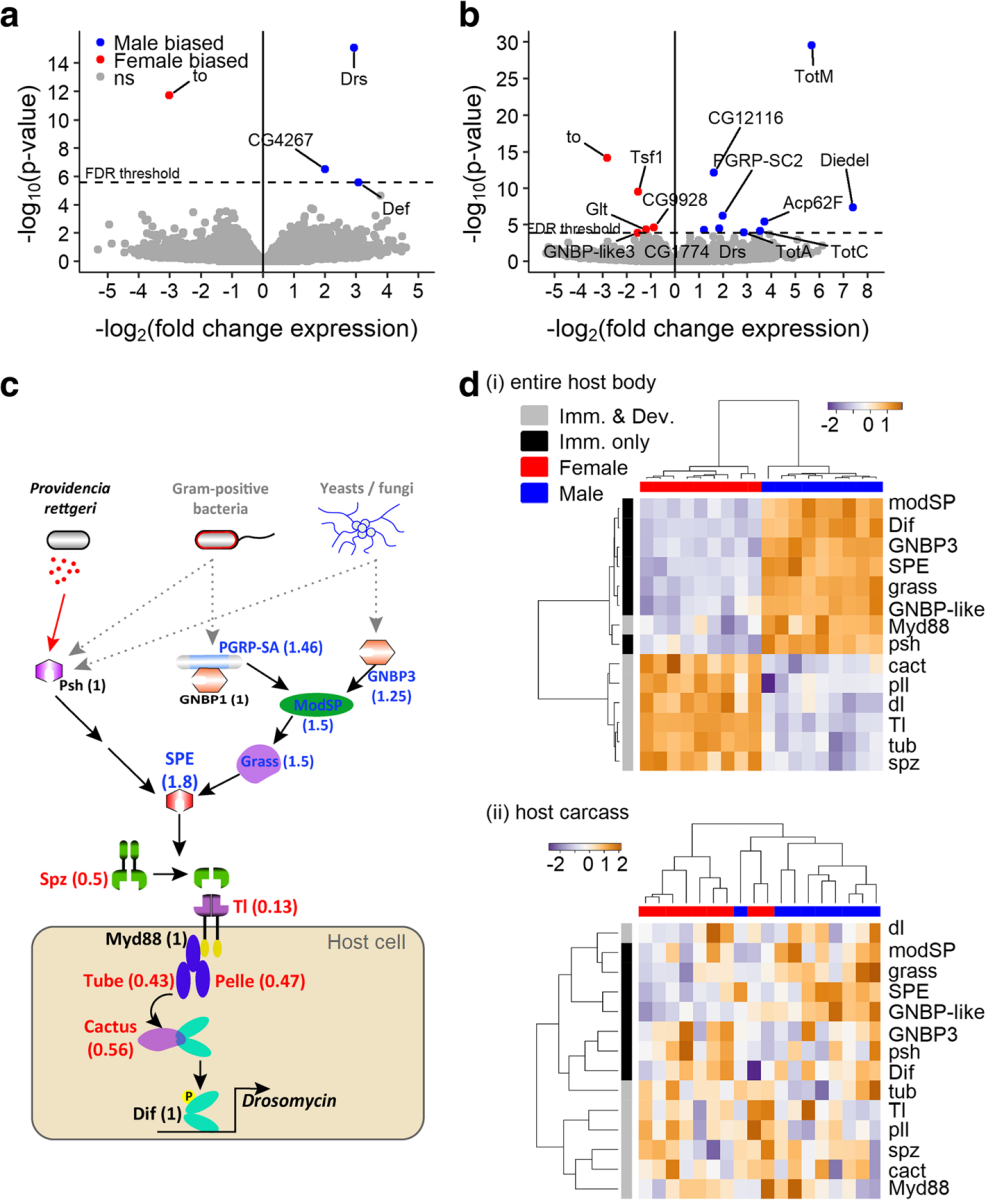
Whole transcriptome response to infection suggest a major role for the constitutive expression of the Toll pathway in the sexual dimorphism. **a** Transcriptional difference in response to infection in whole body of males and females. **b** Transcriptional difference in response to infection in carcasses (i.e., reproductive tissues were removed before RNA collection) of males and females. Those volcano plots represent the genes according to the dimorphism of the response (i.e., interaction *Sex* and *Treatment* in our model) and the significance of the difference. Blue dots are genes from which the response (i.e., difference between unchallenged and infected) is significantly more upregulated in males (or downregulated in females). Red dots are genes from which the response is significantly more upregulated in females (or downregulated in males). Grey dots are non-significant genes after FDR correction for multiple testing. **c** Illustration of the Toll pathway and sexual dimorphism of its constitutive expression based on a publically available meta-analysis [72]. Numbers in brackets represent the male/female ratio of gene expression in whole flies. Males constitutively express genes in the part of the pathway that is specific to the immune function at higher levels than females (part of the pathway outside the host cell, ‘Imm. only’) and females express genes that are used in embryonic development more highly (part of the pathway inside the host cell, ‘Imm. & Dev.’). **d** Constitutive expression of genes belonging to the Toll pathway in nine biological replicates of a pool of 25 non-infected male or female flies. The male-specific constitutive expression of the genes involved only in immunity (black) and the female-specific constitutive expression of the genes shared between immune functions and embryonic development (grey) were confirmed in samples including gene expression in reproductive tissues (i), but disappeared when those tissues were removed by dissection prior to transcriptional analysis (ii). The gradient in the heatmaps represents the level of expression (blue: low and red: high)

A critical step in the activation of the Toll pathway is the cleavage of Spz, which allows it to bind to and activate the Toll receptor. This cleavage can be stimulated by either of two mechanisms [38, 39]. First, recognition of stereotypical cell wall components of fungi and Gram-positive bacteria leads to the proteolytic activation of a Serine protease, ModSP, that initiates a cascade of proteolytic events ultimately leading to Spz processing (the ‘ModSP branch’, see illustration in Fig. 5C) [38]. Alternatively, the Toll pathway can be activated by recognition of virulence factors or host tissue damage through the cleavage of a distinct Serine protease, Persephone, which also leads to the processing of spz (the ‘Psh branch’, see illustration in Fig. 5C) [39, 67, 68]. We asked which branch of the pathway is responsible for Toll-mediated sexual dimorphism by disrupting each branch independently. In *modSP* mutants, females still continue to die significantly more rapidly from *P. rettgeri* infection than males do, although the dimorphism is marginally attenuated (Fig. 4b). In contrast, however, loss-of-function of the *persephone* gene completely abolished the sexual dimorphism in survivorship of *P. rettgeri* infection (Fig. 4c). We confirmed those outcomes using RNAi targeting ModSP and Persephone transcripts (Additional file 6: Figure S6A). Thus, sensing of *P. rettgeri* infection by Persephone allows the recognition of the infection by the Toll pathway and its dimorphic activity ultimately leads to a difference in susceptibility to this infection between males and females.

The Toll pathway is central for the immune response against Gram-positive bacteria [12, 13]. In order to test whether Toll activity is generally dimorphic, we tested whether the Toll pathway also causes dimorphism in response to infection with the Gram-positive bacterium *E. faecalis*. Like *P. rettgeri*, this natural pathogen of *D. melanogaster* induced greater mortality in female than male wildtype hosts (Fig. 1b and Additional file 2: Figure S1C). Female and male mutants for the Toll pathway were equivalently susceptible to *E. faecalis* infection, again indicating that the Toll pathway is required for the immune dimorphism. Furthermore, when we injected male and female flies mutant for either *modSP* or *persephone* with *E. faecalis*, we observed a surprising reversal in the direction of the sexual dimorphism compared to infection in wildtype flies. Males died slightly but significantly earlier than females (Fig. 4d), with males null for *Persephone* dying earlier than males null for *modSP* (Fig. 4d). This finding indicates that in response to *E. faecalis* infection, both the ModSP and Persephone branches have a role in the sexually dimorphic outcome.

Our data suggest that Toll pathway activity or activation could differ between males and females, which we could expect to cause difference in the production of AMPs. We therefore evaluated the expression levels of an antimicrobial peptide gene controlled by Toll (*Drosomycin*) [13]. The induction of *Drosomycin* is much stronger in males than in females at both 24 and 72 h post-injection (Additional file 6: Figure S6B), indicating sustained and dimorphic activation of the Toll pathway in response to the Gram-negative *P. rettgeri* infection. This sexually dimorphic activation was lost in *Psh* and *Spz* mutants but not in *ModSP* mutants (Fig. 4e). The *Spz* mutation eliminated the dimorphism in AMP expression levels more completely than the *Psh* mutation. Our results suggest that the Psh/Toll branch controls the dimorphic expression of the antimicrobial peptide genes.

The role of Psh in the dimorphism in susceptibility reveals that sensing of damage or bacterial virulence is key in the immune dimorphism of *D. melanogaster*. To test whether the observed sexual dimorphism in immunity is conditional on a pathogenic bacterial infection, we evaluated the capacity of Canton S males and females to manage infection with the non-lethal bacteria *E. coli* and *Ecc15* (survival > 95%). These Gram-negative bacteria should activate the Imd pathway but are not predicted to activate the Toll pathway through either the ModSP (peptidoglycan sensing) or Persephone (damage sensing) branches. In accordance with that hypothesis, we saw no difference between males and females in the sustained load of either *Ecc*15 or *E. coli* over a 3-day time course (Additional file 7: Figure S7). These data indirectly support a model where the Persephone/Toll immune cascade that responds to virulence factors is central to *Drosophila* innate immune dimorphism.

### The transcriptional response to infection is sexually dimorphic

Having demonstrated that the Toll pathway is primarily responsible for the sexual dimorphism in response to infection, we next undertook a transcriptomic analysis to identify whether other processes might make secondary contributions to dimorphism. We used RNA-seq to quantify changes in gene expression of males and females at 8 h post-injection, with data taken either from the entire fly or from dissected carcasses in which the reproductive tract and gonads were removed. The resulting data are available and can be browsed on our website (http://flysexsick.buchonlab.com/) or in supplementary Additional file 8: Tables S1 and Additional file 9: Table S2. When expression was quantified from the whole body, we detected four genes for which the response was significantly different between male and female hosts after FDR correction, measured by interaction between *Sex* and *Treatment* in the statistical analysis. These genes are *Drosomycin*, *Defensin*, *CG4267*, and *takeout* (Fig. 5A and Additional file 10: Figure S8A). *Drosomycin* and *Defensin* encode antimicrobial peptides and are downstream of the Toll pathway [13]. The observation that these genes were more highly expressed in males than in females confirms the role of the Toll pathway in establishing dimorphism. *CG4267*, involved in the lipid catabolic process, was also more strongly induced in males. *Takeout*, involved in nutritional homeostasis [69], was downregulated upon infection in males but was not differentially regulated in females.

Because our transcriptional measures were taken from the whole body, apparent differences in the transcriptional response could be due to or masked by differences in gene expression in reproductive tissue between males and females. We therefore performed RNA-seq on the carcasses of males and females from which the reproductive tracts had been dissected away. We again found that *Drosomycin* expression responded more strongly in males than in females (Fig. 5B, Additional file 10: Figure S8B). We additionally confirmed that the male-specific response of *takeout* was independent of the presence of the reproductive tract. In contrast, the dimorphic response of *Defensin* disappeared in the absence of reproductive tissue, indicating that defensin is regulated dimorphically in the reproductive tract with stronger expression in males. The analysis of the dimorphic response of carcasses also revealed additional genes with a dimorphic response to infection that were not identified in whole flies. *Transferrin 1* was more strongly upregulated in females, which suggests a dimorphic role for iron sequestration in the response to infection. Three stress-responsive genes regulated by the Jak-Stat pathway, *TurandotM*, *TurandotA*, and *TurandotC* were strongly induced by infection in males. Even in the absence of reproductive tissue, however, there were few genes with markedly sexually dimorphic regulation (i.e., from which the interaction between *Treatment* and *Sex* was significant). We undertook a GO enrichment analysis where we compared the enrichment of GO categories that are differentially regulated after infection of females with those that are differentially regulated after infection of males. The only GO categories that were then differently enriched between males and females were categories involved in antibacterial humoral response (Tables 1 and 2). This was true regardless of whether the reproductive tract had been removed and supports the interpretation that differences in Toll pathway activity are primarily responsible for the dimorphism in resistance.

**Table 1:** Gene ontology enrichment analysis of gene expression in response to *P. rettgeri* in entire flies

GO term	Description	*P* value^a^	FDR q value^b^	Enrichment (*N*, *B*, *n*, *b*)^c^
GO:0006952	defense response	4.09 × 10^–8^	4.71 × 10^–5^	2.70 (181, 38, 37, 21)
GO:0009617	response to bacterium	2.35 × 10^–7^	1.36 × 10^–4^	2.73 (181, 34, 37, 19)
GO:0043207	response to external biotic stimulus	1.18 × 10^–6^	4.52 × 10^–4^	2.45 (181, 40, 37, 20)
GO:0009607	response to biotic stimulus	1.18 × 10^–6^	3.39 × 10^–4^	2.45 (181, 40, 37, 20)
GO:0051707	response to other organisms	1.18 × 10^–6^	2.71 × 10^–4^	2.45 (181, 40, 37, 20)
GO:0051704	multi-organism process	2.03 × 10^–6^	3.91 × 10^–4^	2.39 (181, 41, 37, 20)
GO:0006955	immune response	2.29 × 10^–6^	3.77 × 10^–4^	2.68 (181, 31, 37, 17)
GO:0009605	response to external stimulus	5.7 × 10^–6^	8.22 × 10^–4^	2.28 (181, 43, 37, 20)
GO:0002376	immune system process	7.72 × 10^–6^	9.9 × 10^–4^	2.52 (181, 33, 37, 17)
GO:0006950	response to stress	1.56 × 10^–5^	1.8 × 10^–3^	2.10 (181, 49, 37, 21)
GO:0042742	defense response to bacterium	3.43 × 10^–5^	3.6 × 10^–3^	2.53 (181, 29, 37, 15)
GO:0050896	response to stimulus	5.73 × 10^–5^	5.51 × 10^–3^	1.81 (181, 65, 37, 24)
GO:0098542	defense response to other organisms	5.94 × 10^–5^	5.28 × 10^–3^	2.45 (181, 30, 37, 15)
GO:0050829	defense response to Gram-negative bacterium	2.8 × 10^–4^	2.31 × 10^–2^	2.88 (181, 17, 37, 10)
GO:0002682	regulation of immune system process	3.07 × 10^–4^	2.36 × 10^–2^	4.19 (181, 7, 37, 6)
GO:0050776	regulation of immune response	3.07 × 10^–4^	2.22 × 10^–2^	4.19 (181, 7, 37, 6)
GO:0031347	regulation of defense response	6.73 × 10^–4^	4.57 × 10^–2^	3.42 (181, 10, 37, 7)
GO:0019731	antibacterial humoral response	9.88 × 10^–4^	6.33 × 10^–2^	2.75 (181, 16, 37, 9)

^a^The enrichment *P* value computed according to the mHG or HG model. This *P* value is not corrected for multiple testing of 1636 GO terms

^b^The correction of the above *P* value for multiple testing using the Benjamini and Hochberg method

^c^Defined as follows: *N* is the total number of genes, *B* is the total number of genes associated with a specific GO term, *n* is the number of genes in the top of the user’s input list or in the target set when appropriate, *b* is the number of genes in the intersection; enrichment = (*b*/*n*)/(*B*/*N*)

**Table 2:** Go enrichment table of gene expression in response to *P. rettgeri* in dissected flies

GO term	Description	*P* value^a^	FDR q value^b^	Enrichment (*N*, *B*, *n*, *b*)^c^
GO:0006955	immune response	4.77 × 10^–5^	7.8 × 10^–2^	1.57 (331, 35, 181, 30)
GO:0050896	response to stimulus	6.02 × 10^–5^	4.92 × 10^–2^	1.31 (331, 95, 181, 68)
GO:0006959	humoral immune response	1.62 × 10^–4^	8.81 × 10^–2^	1.62 (331, 26, 181, 23)
GO:0002376	immune system process	2.44 × 10^–4^	9.99 × 10^–2^	1.49 (331, 38, 181, 31)
GO:0006950	response to stress	3.5 × 10^–4^	1.15 × 10^–1^	1.31 (331, 78, 181, 56)
GO:0009617	response to bacterium	3.69 × 10^–4^	1.01 × 10^–1^	1.45 (331, 43, 181, 34)
GO:0009605	response to external stimulus	4.03 × 10^–4^	9.41 × 10^–2^	1.39 (331, 54, 181, 41)
GO:0043207	response to external biotic stimulus	4.49 × 10^–4^	9.18 × 10^–2^	1.40 (331, 51, 181, 39)
GO:0009607	response to biotic stimulus	4.49 × 10^–4^	8.16 × 10^–2^	1.40 (331, 51, 181, 39)
GO:0051707	response to other organisms	4.49 × 10^–4^	7.34 × 10^–2^	1.40 (331, 51, 181, 39)
GO:0019731	antibacterial humoral response	7.21 × 10^–4^	1.07 × 10^–1^	1.71 (331, 16, 181, 15)

^a^The enrichment *P* value computed according to the mHG or HG model; this *P* value is not corrected for multiple testing of 1636 GO terms

^b^The correction of the above *P* value for multiple testing using the Benjamini and Hochberg method

^c^Defined as follows: *N* is the total number of genes, *B* is the total number of genes associated with a specific GO term, *n* is the number of genes in the top of the user’s input list or in the target set when appropriate, *b* is the number of genes in the intersection; enrichment = (*b*/*n*)/(*B*/*N*)

Sexual dimorphism in resistance to infection could originate from constitutive differences between the sexes prior to infection. We therefore examined the transcriptomic differences between males and females in the absence of infection. We particularly focused on genes related to the Toll pathway. Our hypothesis was that sexual dimorphism in activation of the Toll pathway could be explained by sexually dimorphic constitutive expression of genes encoding components of the Toll signaling cascade, thereby generating dimorphism in immune response. The intracellular signaling component of the Toll pathway is additionally required for dorsoventral patterning of the *Drosophila* embryo in the female ovary [70]. Pattern recognition receptors (e.g., PGRP-SA, GNBP1, GNBP3), as well as serine proteases of the Toll pathway (e.g., Persephone, ModSP, Grass, SPE) are required for the activation of Spz during an immune response (illustrated outside the host cell in Fig. 5C and referred as ‘Imm. only’ in Fig. 5D), whereas Spz is activated by an independent process leading to the serine protease Easter during development [71]. Genes coding for activation of Toll and downstream intracellular signaling participate in both immune and developmental signaling (including *Spaetzle*, *Myd88*, *tube*, *Pelle*, *Cactus*, illustrated in the host cell in Fig. 5C and referred as ‘Imm. & Dev.’ in Fig. 5D). To evaluate the possibility that genes encoding Toll pathway components are expressed constitutively in a dimorphic manner, we undertook two approaches. First, we retrieved the expression levels of these key genes from published transcriptomic datasets obtained from either males or females [72]. These data revealed that genes coding for components of the Toll signaling pathway were constitutively expressed in a sexually dimorphic fashion. Genes from the Toll pathway involved in the immune activation of AMPs were expressed mostly in a male-biased fashion (Ratio M/F expression: *Spe*: 1.8, *grass*: 1.5, *modSP*: 1.5, *GNBP3*: 1.25, *PGRP-SA*: 1.46, *GNBP1*: 1, *persephone*: 1, Fig. 5C). However, downstream genes in the pathway, involved both in immune and germline Toll pathway activity, were significantly female biased (Ratio M/F expression: *spaetzle*: 0.5, *Myd88*: 0.76, *tube*: 0.43, *pelle*: 0.43, Fig. 5C). Second, we evaluated our own expression data from the unchallenged condition (Fig. 5D). We found that sex and involvement in the immune response were primary determinants of hierarchical gene expression clustering (‘Imm. only’ genes vs. ‘Imm. Dev.’ genes in males vs. females Fig. 5Di), confirming the results observed in the meta-analysis. This pattern was, however, not present when the expression in the reproductive tract was removed from the samples (Fig. 5Dii). This suggests strongly that the difference in developmental RNAs are due to the maternal depositions into eggs and are not part of the systemic immune response. Altogether, these results support the hypothesis that the Toll pathway plays a central role in the sexual dimorphism of the response to *P. rettgeri* infection. They also suggest that constitutive sexually dimorphic expression of genes regulating Toll pathway activity is a key determinant of sexual dimorphism in resistance to infection.

## Discussion

Across taxa, studies have shown that disease outcomes are often sexually dimorphic (reviewed in [73]). However, the mechanisms underlying such dimorphism remain poorly characterized. In this study, we show that *D. melanogaster* females are generally less resistant to bacterial infections due to decreased relative activity of the Toll signaling pathway and therefore have a reduced capability of mounting an effective immune response. Drawing from the model proposed by Duneau et al. [50], this reduction in immune inducibility could lead to decreased probability that females control bacterial proliferation in the early stages of infection, thus increasing the probability that they ultimately die from the infection. Although the Toll pathway is generally considered to control Gram-positive bacterial and fungal infections, we found it to be responsible for observed dimorphism in susceptibility to both the Gram-positive bacterium *E. faecalis* and the Gram-negative *P. rettgeri*. We note that this dimorphism in Toll activity effectively results in male and female hosts being different ‘environments’ from the perspective of the parasite, with potential consequence for the evolution of pathogens that have sex-biased transmission.

### What makes males and females dimorphic

The activation of the Toll pathway is triggered downstream of either the recognition of bacterial cell wall components (ModSP branch) or the detection of proteolytic activity in hemolymph (Persephone branch) (Fig. 5C). *Persephone* has been proposed to also mediate Toll activation downstream of endogenous danger signals [39]. The requirement of the Persephone branch for the increased male survivorship of *P. rettgeri* infection suggest two hypotheses. On the one hand, it is possible that the dimorphic response is directly due to a dimorphism in recognition of danger signals or virulence factors through Persephone-mediated sensing. On the other, it is also possible that the Persephone branch is not itself dimorphic, but that its activation by pathogenic infection exposes inherent dimorphism in downstream components of the Toll pathway; the latter hypothesis seems more likely. The genes of the Toll pathway that are specific to the immune system are constitutively male-biased in their expression (Fig. 5C, D), supporting the idea that once the recognition occurs via the Persephone branch, the Toll pathway can respond more strongly in males, leading to a higher expression of the AMPs. Furthermore, it is also possible that, in addition to this dimorphism in constitutive expression, a dimorphism in activation of the pathway during the infection may translate into subsequent differences between sexes.

### Proximal cause for the immune sexual dimorphism

Although the Toll pathway is clearly dimorphic between males and females, the basis for this dimorphism remains to be determined. One reasonable hypothesis is that hormonal differences between females and males generate the difference in Toll activity. The importance of juvenile hormone (JH) in oogenesis and in female response to mating supports the hypothesis that males and females may have different levels of circulating JH. JH has been shown to downregulate the immune system with a stronger influence on the Toll pathway than on the Imd pathway (as shown by Figure 1 of Flatt et al. [74]). Mating and the transfer of male seminal fluid proteins affects the female innate system [75], stimulating the production of JH in female *D. melanogaster* [76, 77] and depressing immune performance in mated females relative to virgins [23, 24]. Since we found dimorphism even between virgin females and mated males, it is possible that the dimorphism in the expression of the Toll pathway is the result of dimorphic levels of JH in females even before mating. The immunosuppressant effect of hormones is frequently proposed to be the main proximal mechanism for sexual dimorphism in vertebrate susceptibility to infection [78], although studies on mammals have emphasized the acquired immune system with little attention to the innate immune system. Based on the *Drosophila* data, we propose that endocrinologic differences may also influence sexual dimorphism in innate immunity.

### Evolutionary consequences of immune sexual dimorphism

From the perspective of an infecting pathogen, male and female hosts may be different ‘environments’ with, among other variations, different levels of immune activity. It is thus reasonable to assume that parasite lineages could adapt to these distinct environments either when transmission is sex biased, either due to unequal host sex-ratio, sex-based social structure, or endogenous susceptibility of one host sex relative to the other [70]. Furthermore, dimorphism in host resistance, as is the case against *P. rettgeri* infections, imposes a specific kind of selection pressure on pathogens. Theory predicts that hosts with a strong immune response select for more virulent parasites with a higher transmission. In the case of extreme difference in resistance, evolution of increased virulence in one sex may result in a parasite that kills the non-resistant sex so quickly that the pathogen can persist and evolve only in the resistant host [79]. In the case of sexual dimorphism in host resistance, as observed in our study, sex-specific adaptation of parasites is therefore expected to evolve [4, 73]. Our results show that *D. melanogaster* can have a strong sexual dimorphism in resistance to certain bacterial infections and can therefore be a good model to explore the consequences of sex-specific adaptation on sex-specific symptoms and prevalence of infectious diseases.

From the host perspective, females are expected to invest more in immunity in order to maximize lifetime reproduction (Bateman’s principle of immunity) or because of the direct negative interaction between reproductive and immune traits (immunocompetence handicap hypothesis) [80, 81]. These simplified evolutionary models have often led to the prediction that animals should have an immunologically ‘weaker sex’ [5], and tend to ignore the variability that has already been highlighted. Indeed, males from mammalian species are generally more susceptible to infectious diseases like leishmaniasis, malaria, and schistosomiasis and females to toxoplasmosis, amoebiasis, and giardiasis [6, 78, 82]. In *D. melanogaster*, the observation that males were more susceptible than females to *S. aureus* infections highlights the parasite dependence of sexual dimorphism in immunity. The sex-specific investment in defense is probably the result of independent investments in different functions of the immune system (e.g., humoral and cellular responses) linked to sex-specific constraints or pleiotropy, and not of a general weakness [61]. Indeed, in *Drosophila*, *S. aureus* infection is generally controlled by phagocytosis and melanization and the recognition of *S. aureus* infection by the Toll pathway can be limited [31, 83, 84]. Which sex has higher relative susceptibility may therefore depend on the identity of the infecting parasite, exposing difficulties in drawing generalizations about the sexual dimorphism via meta-analyses [85]. As we begin to consider personalized medicine and bridging the ‘gender-gap’ in biomedical studies as well as the role of host sex in epidemiology, it appears increasingly important to consider, in specific details, the host dimorphism in response to infection.

## Additional files

Additional file 1: Figure S4. Sexual dimorphism in phagocytosis. (A) Confirmation for reduced number of phagocytes in Hml-bax lines. Females from Hml-GFP lines also had more phagocytes than males (Welsh *t* test: df = 6.66, *t* = 2.53, *P* = 0.04). The depletion of phagocytes in Hml-bax was major and removed the sexual dimorphism in counts (Welsh *t* test: df = 8.13, *t* = 0.86, *P* = 0.41). (B) Illustration by confocal microscopy of phagocytosis by *P. rettgeri*. Host hemocytes constitutively expressed red fluorescence, while the bacteria constitutively expressed green fluorescence. The overlay of the two color channels (red and green) shows that *P. rettgeri* was phagocytized by the host. (C) Left panel: Counts of phagocytes for pool of 15 females or males. Females have more phagocytes than males (Wilcoxon paired test: n = 8 per lines, V = 238, *P* = 0.002). One estimate for an *Eater* female of 1800 hemocytes and was not represented in the figure for a better display of the whole dataset. Right panel: Counts of phagocytes that phagocytized 3 h post-injection of dead *E. coli*. The number of active phagocytes upon injection of dead Gram-negative bacteria did not significantly differ between the sexes (Linear model (Sex being nested in experimental trials), *Sex*: df = 1, F = 0.89, *P* = 0.44). (TIF 221 kb)

Additional file 2: Figure S1. Sexual dimorphism in survival to infection of inbred populations. (A) Survival of the genotype Canton S upon infection by the Gram-negative bacteria *P. alcalifaciens*. Females were more susceptible than males (Cox-ph: *Sex*: df = 1, χ^2^ = 11.94, *P* = 0.0005). (B) Dose response in survival of the genotype Oregon R to *P. rettgeri* infection. Females were more susceptible than males (Cox-ph: *Sex*: df = 1, χ^2^ = 15.95, *P* < 0.0001) and the dose had an effect on survival (*Dose*: df = 1, χ^2^ = 16.59, *P* = 0.0002) but this effect was dependent on the host sex (Dose*Sex: df = 1, χ^2^ = 6.13, *P* = 0.04). (C) Dose response in survival of Canton S to the Gram-positive *E. faecalis*. Females were more susceptible than males (Cox-ph: *Sex*: df = 1, χ^2^ = 29.061, *P* < 0.0001) and the dose had an effect on survival (*Dose*: df = 1, χ^2^ = 16.51, *P* < 0.0001), but we did not detect a difference in response to the dose (*Dose*/*Sex*: df = 1, χ^2^ = 0.26, *P* = 0.61). (TIF 223 kb)

Additional file 3: Figure S2. Sexual dimorphism in total mass and hemolymph quantity. (A) In the three wildtype lines, the total mass differs between lines but less than between sexes (two-way ANOVA: Line: df = 2, F = 275,92, *P* < 0.0001; Sex: df = 1, F = 558.11, *P* < 0.0001; Line*Sex: df = 2, F = 33.88, *P* < 0.0001). (B) In the three wildtype lines, the quantity of hemolymph differs among lines but less than between sexes (two-way ANOVA: Line: df = 2, F = 18.65, *P* < 0.0001; Sex: df = 1, F = 69.33, *P* < 0.0001; Line*Sex: df = 2, F = 3.84, *P* = 0.02). Females are 1.5 times larger than males at most, which is insufficient to explain the difference in bacterial load between males and females (Fig. 2) or the observation that males are resistant to 10-times larger initial does than females (Fig. 1i, Additional file 2: Figure S1B, C). (TIF 155 kb)

Additional file 4: Figure S3. Sexual dimorphism in bacterial growth in dead host. *P. rettgeri* growth was sexually dimorphic in living Canton S flies (*Time*: df = 1, F = 54.35, *P* < 0.001; *Sex*: df = 1, F = 11.27, *P* = 0.001; *Time:Sex*: df = 1, F = 7.55, *P* = 0.008). However, we did not detect significant differences in bacterial load over time when hosts were killed by decapitation 1 h before or at the moment of the injection (Killed before: *Time*: df = 1, F = 131.8, *P* < 0.001; *Sex*: df = 1, F = 0.09, *P* = 0.75, *Time/Sex*: df = 1, F = 2.86, *P* = 0.1; Killed after: *Time*: df = 1, F = 63, *P* < 0.001; *Sex*: df = 1, F = 0.03, *P* = 0.86; *Time:Sex*: df = 1, F = 0.0001, *P* = 0.99). Our results suggest that males and females do not differ in resources available to the bacteria but do differ in capacity to actively control infection. (TIF 149 kb)

Additional file 5: Figure S5. Sexual dimorphism of the Imd pathway. (A) *dTak1* loss-of-function mutants are also sexually dimorphic (Cox-ph: *Sex*: df = 1, χ^2^ = 44.2, *P* < 0.0001). (B) Males and females with a *Relish* loss-of-function mutation are still sexually dimorphic in bacterial load (Welsh *t* test: at 8 h: df = 2.15, *t* = 19.33, *P* = 0.04; at 12 h: df = 2.25, *t* = 17.26, *P* = 0.04). (TIF 160 kb)

Additional file 6: Figure S6. Sexual dimorphism of the Toll pathway. (A) Survival of male and female with downregulation of *modSP* (da-Gal4 > modSP-RNAi), of *psh* (*da-Gal4 > psh-RNAi*) or of wildtype (*da-Gal4 > Canton S*). We confirmed via RNA interference that psh but not modSP was responsible for the sexual dimorphism in survival upon *P. rettgeri* infection (Cox-ph: *Line* (psh vs. Canton S): df = 1, χ^2^ = 0.22, *P* = 0.63, *Sex*: df = 1, χ^2^ = 13.97, *P* = 0.0002, Line*Sex: df = 1, χ^2^ = 0.73, *P* = 0.4; Cox-ph: *Line* (modSP vs. Canton S): df = 1, χ^2^ = 2.14, *P* = 0.14, *Sex*: df = 1, χ^2^ = 8.18, *P* = 0.004, Line*Sex: df = 1, χ^2^ = 4.23, *P* = 0.04). (B) Relative expression of *Drosomycin* to *RpL32* in both sexes 24 and 72 h after the injection of PBS or of *P. rettgeri*. Toll response (i.e., difference in *Drosomycin* expression between PBS and infected) was stronger in male than in females at 72 h (Interaction *Sex* x *Treatment*: df = 1, F = 10.64, *P* = 0.006) but we did not detect a difference at 24 h post-injection (Interaction *Sex* x *Treatment*: df = 1, F = 2.53, *P* = 0.13). (TIF 180 kb)

Additional file 7: Figure S7. Sexual dimorphism in clearance of Gram-negative non-pathogenic bacteria. Male and female Canton S flies are similarly capable of resisting the bacteria *Ecc15* (linear regression: *Time*: df = 2, F = 4.29, *P* = 0.02; *Sex*: df = 1, F = 2.46, *P* = 0.12; *Time/Sex*: df = 2, F = 3.02, *P* = 0.05) and *E. coli* (Linear regression: *Time*: df = 2, F = 5.31, *P* = 0.006; *Sex*: df = 1, F = 0.0001, *P* = 0.99; *Time/Sex*: df = 2, F = 0.12, *P* = 0.89). Non-pathogenic Gram-negative bacteria are not differently controlled by male and female hosts. (TIF 161 kb)

Additional file 8: Table S1. Count matrix for the transcriptomic analysis. This matrix contains the normalized read counts used in the DESeq2 analysis with the function “*DESeqDataSetFromMatrix*”. (CSV 2291 kb)

Additional file 9: Table S2. Column information for the transcriptomic analysis. This table contains the information on the samples used in the DESeq2 analysis with the function “*DESeqDataSetFromMatrix*” and Additional file 8: Table S1. (CSV 1 kb)

Additional file 10: Figure S8. Sexual dimorphism in expression of genes significantly different in RNAseq experiments. (A) Number of reads per million (RPM) of genes responding differently in males (blue) and females (red) upon infection with *P. rettgeri*. (B) Number of RPM of genes responding differently in tissues, except reproductive tissues, of males (blue) and females (red) upon infection with *P. rettgeri*. The shape of the dots represents the replicated experiments and each dot is the expression quantified in a pool of 25 flies. Black bars represent the means of the three replicated experiments. (TIF 1614 kb)
