# Supplementary figures and images for: The Toll pathway underlies host sexual dimorphism in resistance to both Gram-negative and Gram-positive bacteria in mated Drosophila
Source: BMC Biol. 2017 Dec 21;15:124. doi: 10.1186/s12915-017-0466-3 (PMC5740927; doi:10.1186/s12915-017-0466-3)

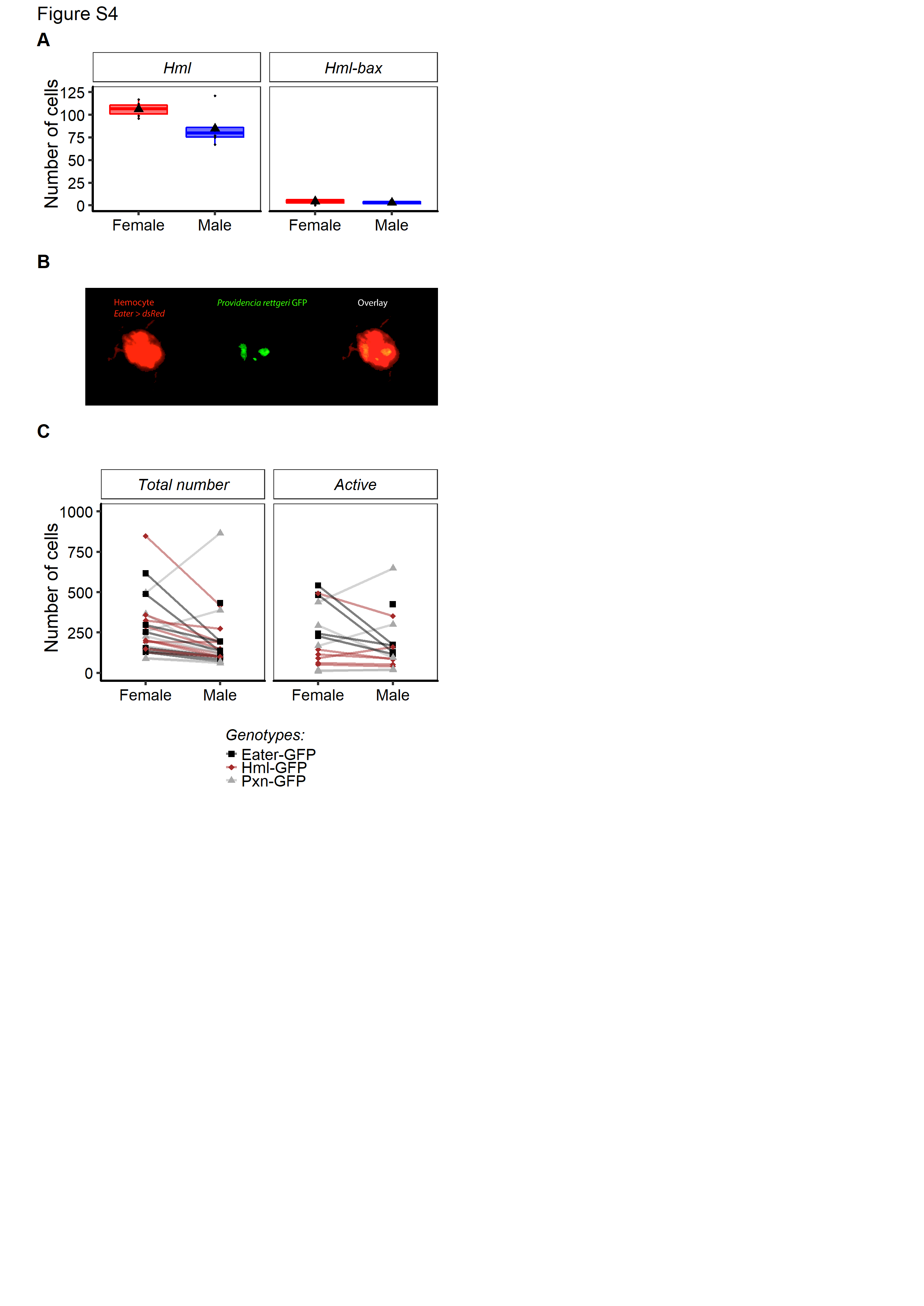

Supplement: Supplementary file 1 — Sexual dimorphism in phagocytosis. (A) Confirmation for reduced number of phagocytes in Hml-bax lines. Females from Hml-GFP lines also had more phagocytes than males (Welsh t test: df = 6.66, t = 2.53, P = 0.04). The depletion of phagocytes in Hml-bax was major and removed the sexual dimorphism in counts (Welsh t test: df = 8.13, t = 0.86, P = 0.41). (B) Illustration by confocal microscopy of phagocytosis by P. rettgeri. Host hemocytes constitutively expressed red fluorescence, while the bacteria constitutively expressed green fluorescence. The overlay of the two color channels (red and green) shows that P. rettgeri was phagocytized by the host. (C) Left panel: Counts of phagocytes for pool of 15 females or males. Females have more phagocytes than males (Wilcoxon paired test: n = 8 per lines, V = 238, P = 0.002). One estimate for an Eater female of 1800 hemocytes and was not represented in the figure for a better display of the whole dataset. Right panel: Counts of phagocytes that phagocytized 3 h post-injection of dead E. coli. The number of active phagocytes upon injection of dead Gram-negative bacteria did not significantly differ between the sexes (Linear model (Sex being nested in experimental trials), Sex: df = 1, F = 0.89, P = 0.44). (TIF 221 kb) [file 12915_2017_466_MOESM1_ESM.tif]

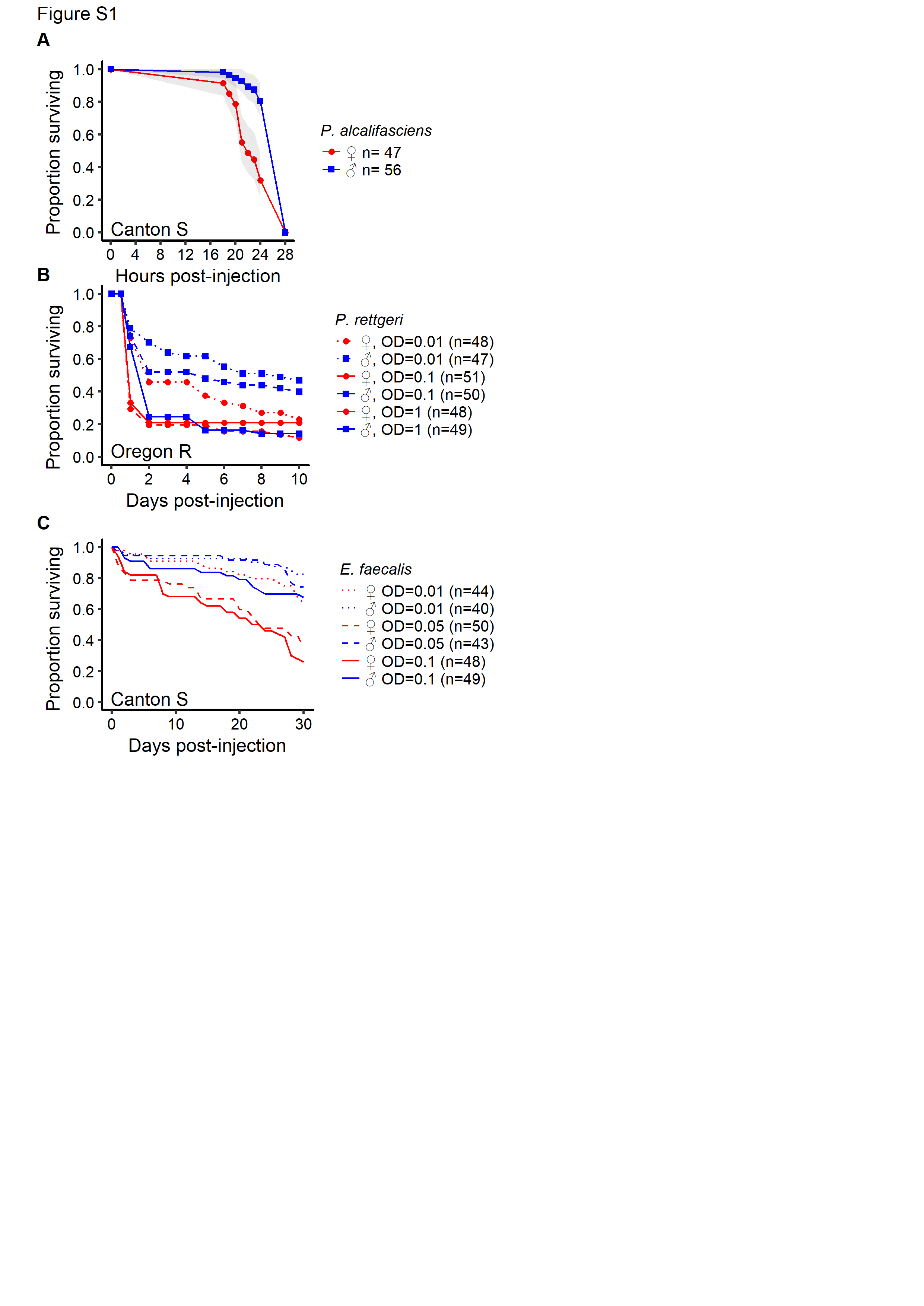

Supplement: Supplementary file 2 — Sexual dimorphism in survival to infection of inbred populations. (A) Survival of the genotype Canton S upon infection by the Gram-negative bacteria P. alcalifaciens. Females were more susceptible than males (Cox-ph: Sex: df = 1, χ2 = 11.94, P = 0.0005). (B) Dose response in survival of the genotype Oregon R to P. rettgeri infection. Females were more susceptible than males (Cox-ph: Sex: df = 1, χ2 = 15.95, P < 0.0001) and the dose had an effect on survival (Dose: df = 1, χ2 = 16.59, P = 0.0002) but this effect was dependent on the host sex (Dose*Sex: df = 1, χ2 = 6.13, P = 0.04). (C) Dose response in survival of Canton S to the Gram-positive E. faecalis. Females were more susceptible than males (Cox-ph: Sex: df = 1, χ2 = 29.061, P < 0.0001) and the dose had an effect on survival (Dose: df = 1, χ2 = 16.51, P < 0.0001), but we did not detect a difference in response to the dose (Dose/Sex: df = 1, χ2 = 0.26, P = 0.61). (TIF 223 kb) [file 12915_2017_466_MOESM2_ESM.tif]

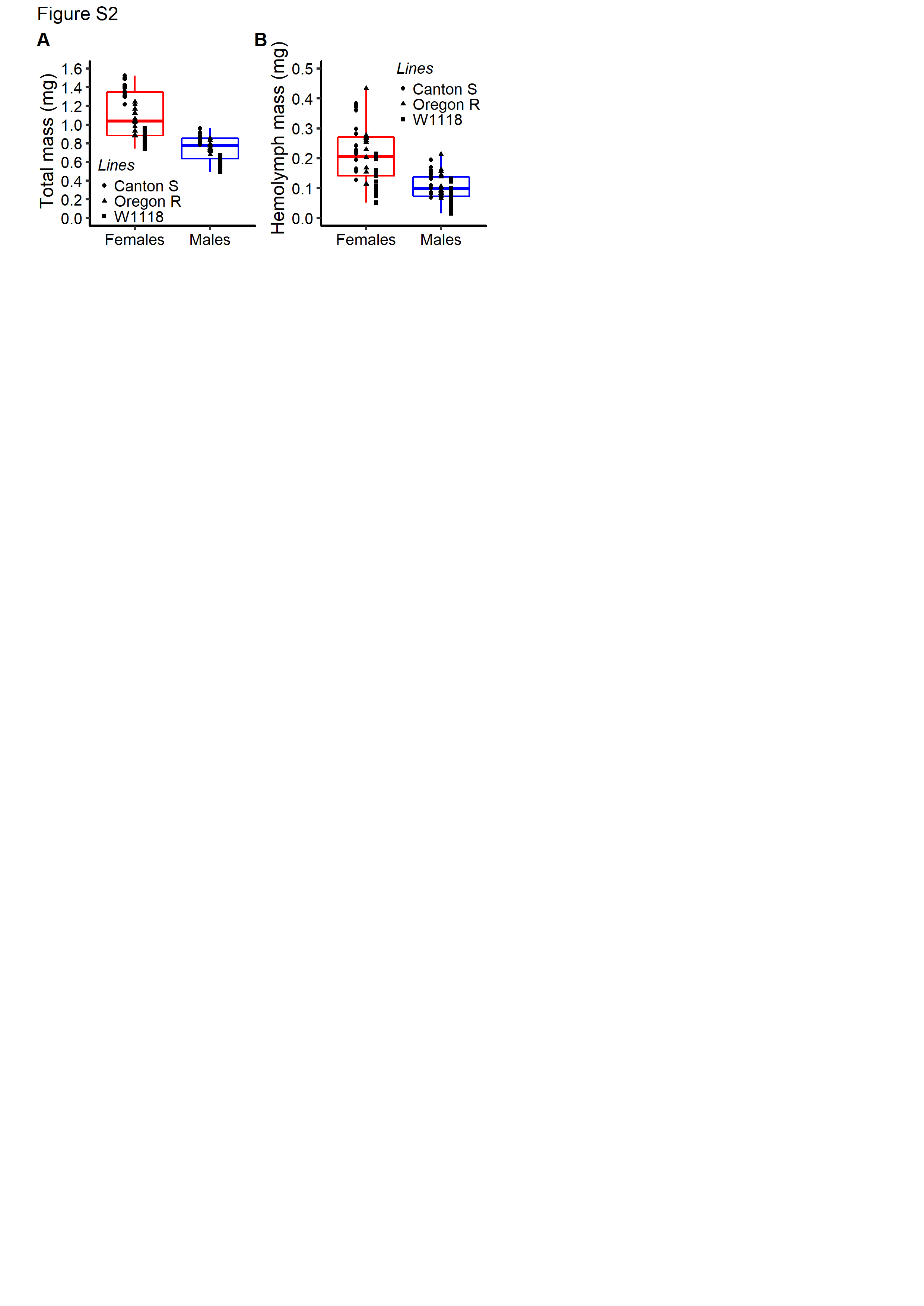

Supplement: Supplementary file 3 — Sexual dimorphism in total mass and hemolymph quantity. (A) In the three wildtype lines, the total mass differs between lines but less than between sexes (two-way ANOVA: Line: df = 2, F = 275,92, P < 0.0001; Sex: df = 1, F = 558.11, P < 0.0001; Line*Sex: df = 2, F = 33.88, P < 0.0001). (B) In the three wildtype lines, the quantity of hemolymph differs among lines but less than between sexes (two-way ANOVA: Line: df = 2, F = 18.65, P < 0.0001; Sex: df = 1, F = 69.33, P < 0.0001; Line*Sex: df = 2, F = 3.84, P = 0.02). Females are 1.5 times larger than males at most, which is insufficient to explain the difference in bacterial load between males and females (Fig. 2) or the observation that males are resistant to 10-times larger initial does than females (Fig. 1i, Additional file 2: Figure S1B, C). (TIF 155 kb) [file 12915_2017_466_MOESM3_ESM.tif]

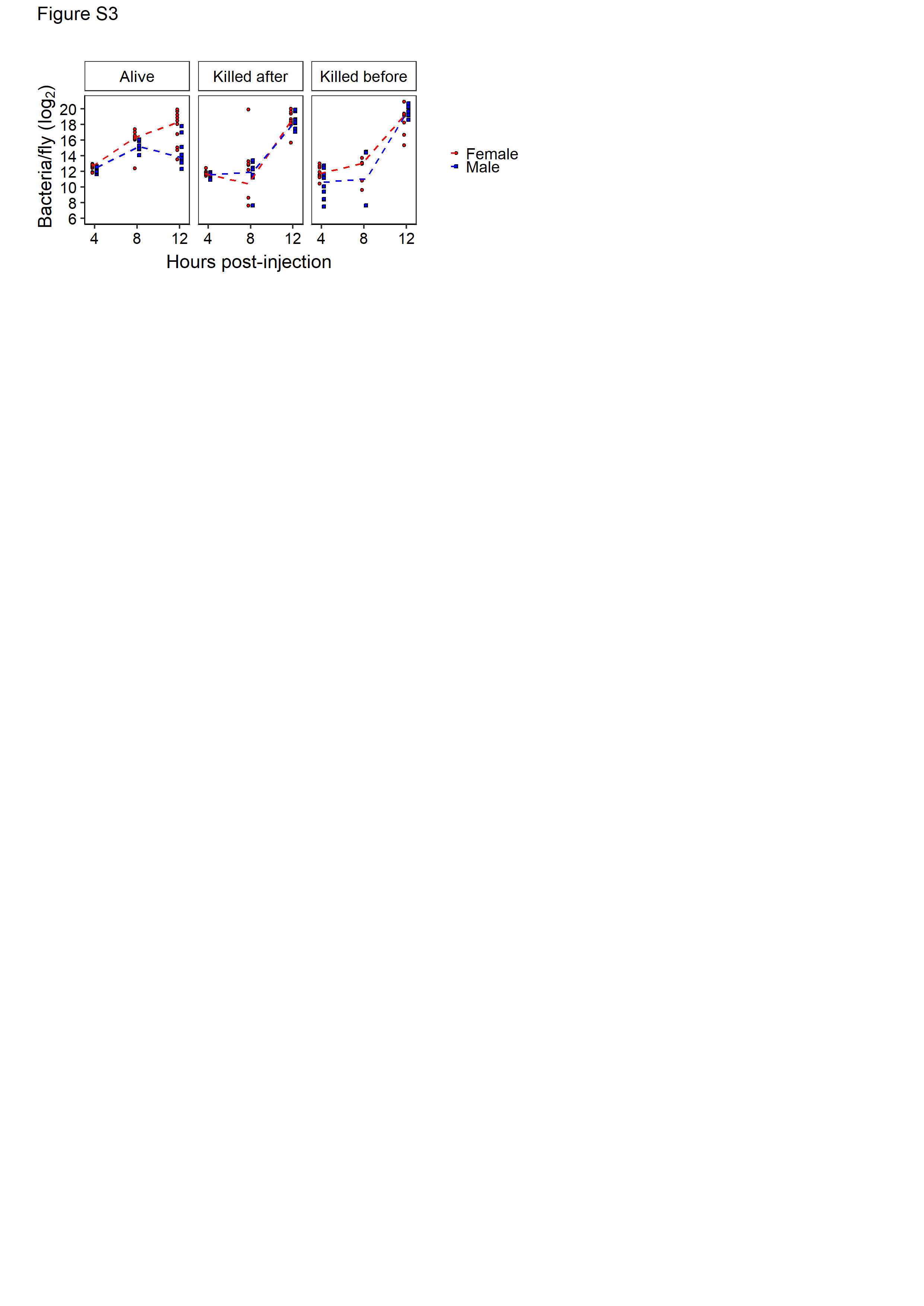

Supplement: Supplementary file 4 — Sexual dimorphism in bacterial growth in dead host. P. rettgeri growth was sexually dimorphic in living Canton S flies (Time: df = 1, F = 54.35, P < 0.001; Sex: df = 1, F = 11.27, P = 0.001; Time:Sex: df = 1, F = 7.55, P = 0.008). However, we did not detect significant differences in bacterial load over time when hosts were killed by decapitation 1 h before or at the moment of the injection (Killed before: Time: df = 1, F = 131.8, P < 0.001; Sex: df = 1, F = 0.09, P = 0.75, Time/Sex: df = 1, F = 2.86, P = 0.1; Killed after: Time: df = 1, F = 63, P < 0.001; Sex: df = 1, F = 0.03, P = 0.86; Time:Sex: df = 1, F = 0.0001, P = 0.99). Our results suggest that males and females do not differ in resources available to the bacteria but do differ in capacity to actively control infection. (TIF 149 kb) [file 12915_2017_466_MOESM4_ESM.tif]

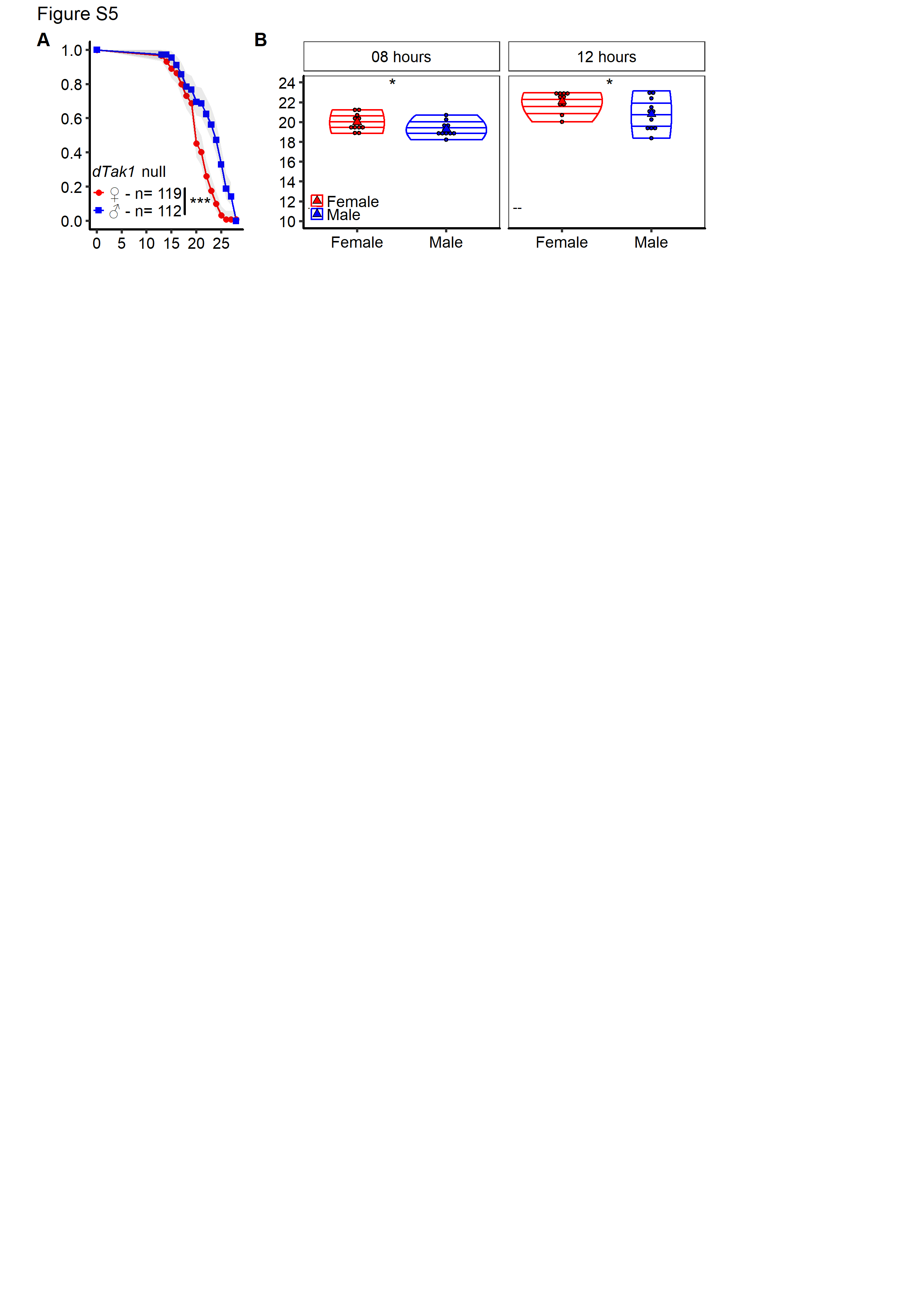

Supplement: Supplementary file 5 — Sexual dimorphism of the Imd pathway. (A) dTak1 loss-of-function mutants are also sexually dimorphic (Cox-ph: Sex: df = 1, χ2 = 44.2, P < 0.0001). (B) Males and females with a Relish loss-of-function mutation are still sexually dimorphic in bacterial load (Welsh t test: at 8 h: df = 2.15, t = 19.33, P = 0.04; at 12 h: df = 2.25, t = 17.26, P = 0.04). (TIF 160 kb) [file 12915_2017_466_MOESM5_ESM.tif]

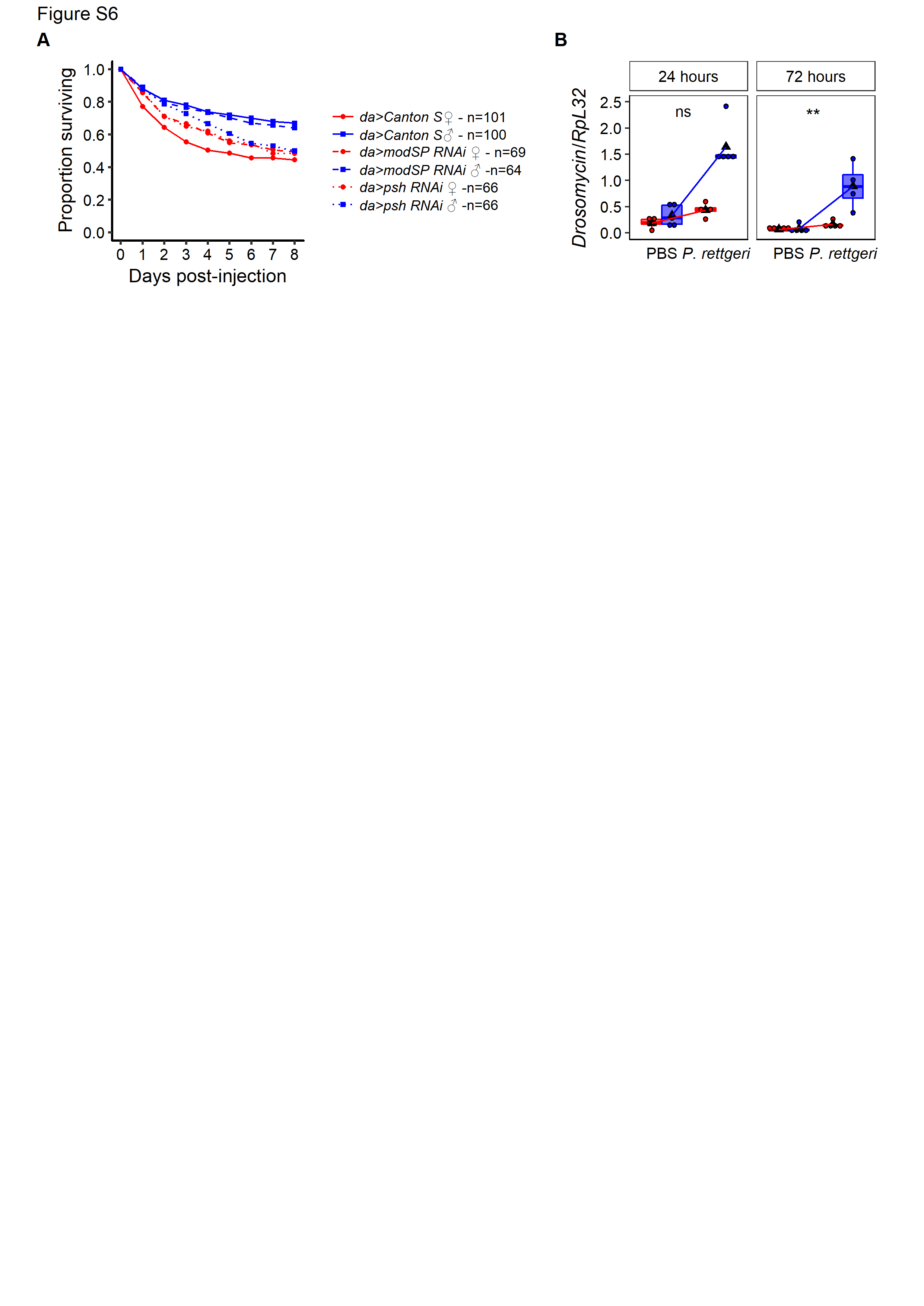

Supplement: Supplementary file 6 — Sexual dimorphism of the Toll pathway. (A) Survival of male and female with downregulation of modSP (da-Gal4 > modSP-RNAi), of psh (da-Gal4 > psh-RNAi) or of wildtype (da-Gal4 > Canton S). We confirmed via RNA interference that psh but not modSP was responsible for the sexual dimorphism in survival upon P. rettgeri infection (Cox-ph: Line (psh vs. Canton S): df = 1, χ2 = 0.22, P = 0.63, Sex: df = 1, χ2 = 13.97, P = 0.0002, Line*Sex: df = 1, χ2 = 0.73, P = 0.4; Cox-ph: Line (modSP vs. Canton S): df = 1, χ2 = 2.14, P = 0.14, Sex: df = 1, χ2 = 8.18, P = 0.004, Line*Sex: df = 1, χ2 = 4.23, P = 0.04). (B) Relative expression of Drosomycin to RpL32 in both sexes 24 and 72 h after the injection of PBS or of P. rettgeri. Toll response (i.e., difference in Drosomycin expression between PBS and infected) was stronger in male than in females at 72 h (Interaction Sex x Treatment: df = 1, F = 10.64, P = 0.006) but we did not detect a difference at 24 h post-injection (Interaction Sex x Treatment: df = 1, F = 2.53, P = 0.13). (TIF 180 kb) [file 12915_2017_466_MOESM6_ESM.tif]

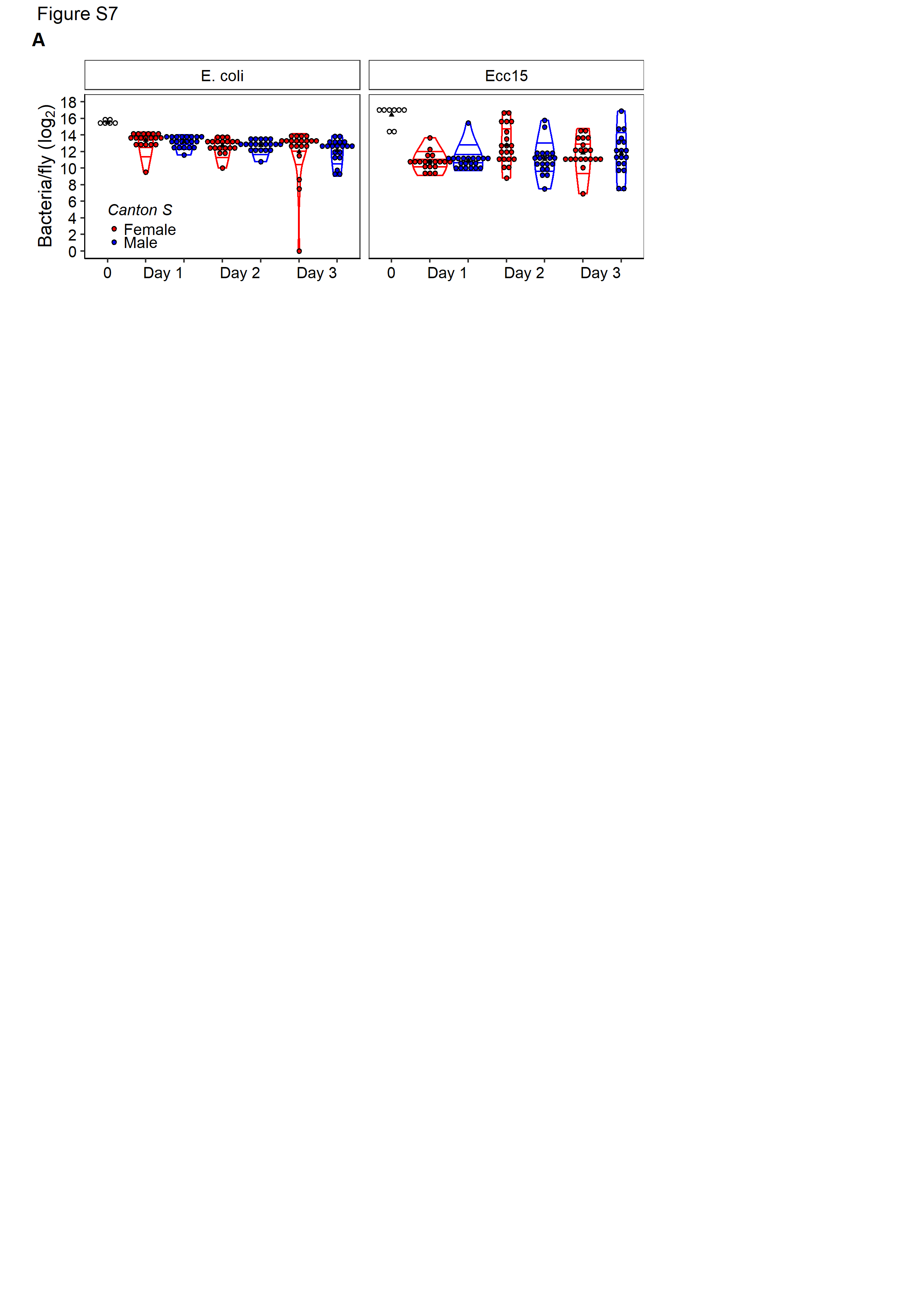

Supplement: Supplementary file 7 — Sexual dimorphism in clearance of Gram-negative non-pathogenic bacteria. Male and female Canton S flies are similarly capable of resisting the bacteria Ecc15 (linear regression: Time: df = 2, F = 4.29, P = 0.02; Sex: df = 1, F = 2.46, P = 0.12; Time/Sex: df = 2, F = 3.02, P = 0.05) and E. coli (Linear regression: Time: df = 2, F = 5.31, P = 0.006; Sex: df = 1, F = 0.0001, P = 0.99; Time/Sex: df = 2, F = 0.12, P = 0.89). Non-pathogenic Gram-negative bacteria are not differently controlled by male and female hosts. (TIF 161 kb) [file 12915_2017_466_MOESM7_ESM.tif]

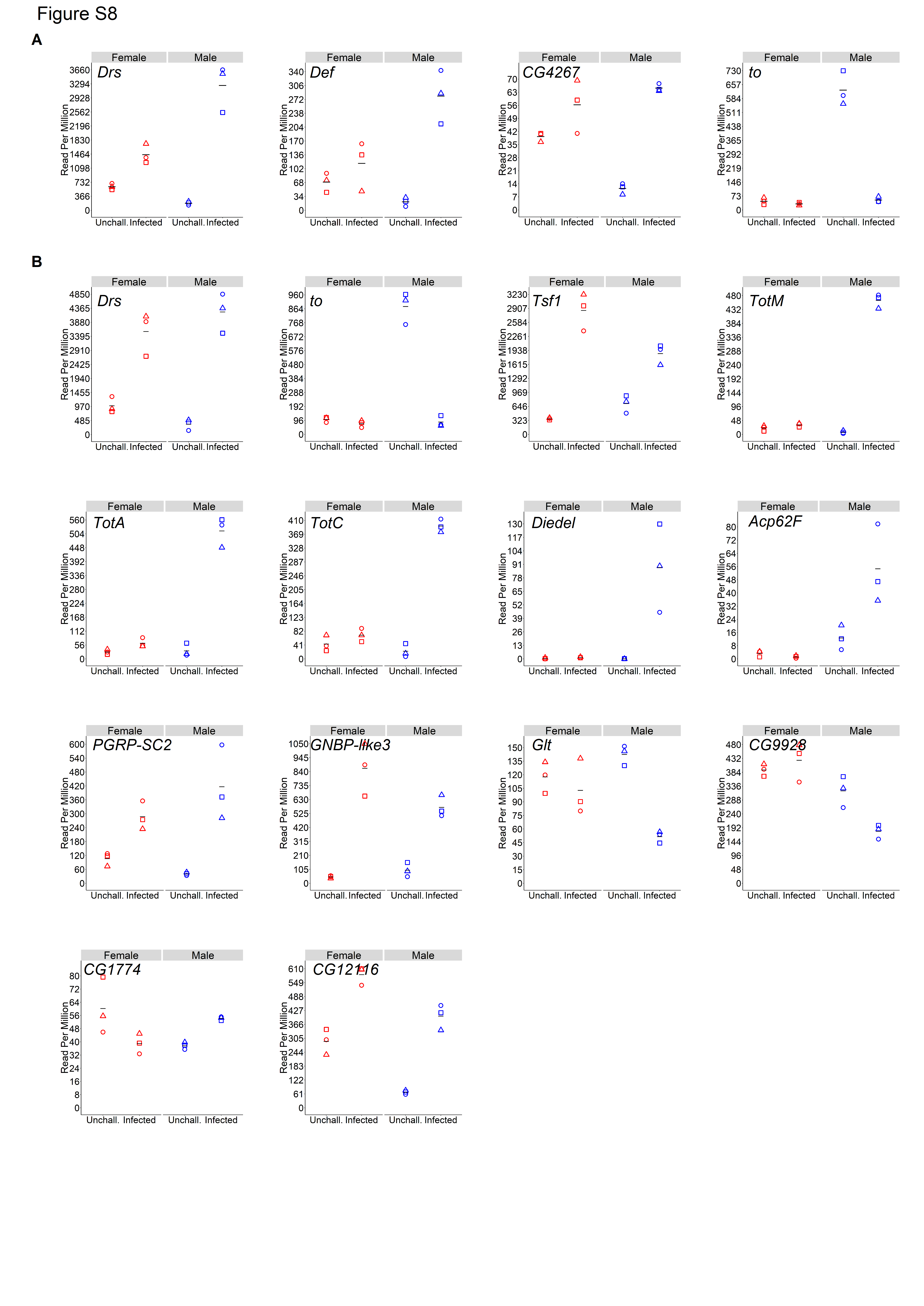

Supplement: Supplementary file 10 — Sexual dimorphism in expression of genes significantly different in RNAseq experiments. (A) Number of reads per million (RPM) of genes responding differently in males (blue) and females (red) upon infection with P. rettgeri. (B) Number of RPM of genes responding differently in tissues, except reproductive tissues, of males (blue) and females (red) upon infection with P. rettgeri. The shape of the dots represents the replicated experiments and each dot is the expression quantified in a pool of 25 flies. Black bars represent the means of the three replicated experiments. (TIF 1614 kb) [file 12915_2017_466_MOESM10_ESM.tif]
